# Circadian patterns of device-detected postoperative atrial fibrillation during 2.5 years of continuous monitoring after cardiac surgery: The RACE V–Tissue Bank Study

**DOI:** 10.1016/j.hroo.2026.06.023

**Published:** 2026-07-03

**Authors:** Michal J. Kawczynski, Martijn D. Gilbers, Joris Winters, Bart Maesen, Aaron Isaacs, Dominik Linz, Michiel Rienstra, Isabelle van Gelder, Jos G. Maessen, Elham Bidar, Ulrich Schotten

**Affiliations:** 1 Department of Cardiothoracic Surgery, Heart and Vascular Centre Maastricht University Medical Centre, Maastricht, The Netherlands; 2 Department of Physiology, Maastricht University, Maastricht, The Netherlands; 3 Cardiovascular Research Institute Maastricht (CARIM), Maastricht, The Netherlands; 4 Maastricht Centre for Systems Biology (MaCSBio), Maastricht University, Maastricht, The Netherlands; 5 Department of Cardiology, Heart and Vascular Centre Maastricht University Medical Centre, Maastricht, The Netherlands; 6 Department of Biomedical Sciences, Faculty of Health and Medical Sciences, University of Copenhagen, Copenhagen, Denmark; 7 Department of Cardiology, University of Groningen, University Medical Centre Groningen, Groningen, The Netherlands; 8 Department of Cardiothoracic surgery, Medisch Spectrum Twente, Enschede, The Netherlands

**Keywords:** Atrial fibrillation, Cardiac surgery, Circadian patterns, Implantable loop recorder, Autonomic regulation

## Abstract

**Background:**

Postoperative atrial fibrillation (POAF) is common after cardiac surgery. AF in non-surgical patients exhibits distinct circadian patterns (adrenergic/daytime and vagal/nocturnal), but such patterns for new-onset POAF and recurrent AF remain undefined.

**Objective:**

This study aimed to characterize circadian AF patterns and relate chronotypes to clinical, biochemical, histological, and outcome features.

**Methods:**

Patients undergoing first cardiac surgery, with and without preoperative AF, were continuously monitored for 2.5 years with an implantable loop recorder. Early AF was defined as AF within 30 days. Patients with sustained AF (≥20 episodes) were chronotyped via circular statistics and local regression of episode duration and ventricular rate (Vrate), and classified as adrenergic, vagal, mixed, or non-rhythmic. Preoperative imaging, electrocardiography, biomarkers, and paired atrial histology were compared across chronotypes.

**Results:**

Of the 98 patients (71 without and 27 with preoperative AF), 53 (54.1%) developed POAF (3607 episodes). Patients with prior AF showed a morning onset peak, whereas new-onset POAF showed a uniform 24-hour distribution. Of 26 patients with sustained AF, 20 were rhythmic (9 adrenergic, 7 vagal, 4 mixed), and 6 were non-rhythmic; 27 had incidental AF (<20 episodes). Rhythmic AF correlated with larger atrial volumes, prolonged PR intervals, higher bone morphogenetic protein-10 levels, and greater right-atrial endomysial fibrosis and asymmetry, most pronounced in the mixed chronotype. Adrenergic AF was associated with more arrhythmia-related emergency visits and cardioversions.

**Conclusion:**

AF after cardiac surgery comprises distinct circadian subtypes. New-onset POAF and recurrent AF display different circadian patterns, and individual chronotypes are associated with varying degrees of atrial remodeling and clinical outcomes.

## Graphical Abstract

**Figure:**
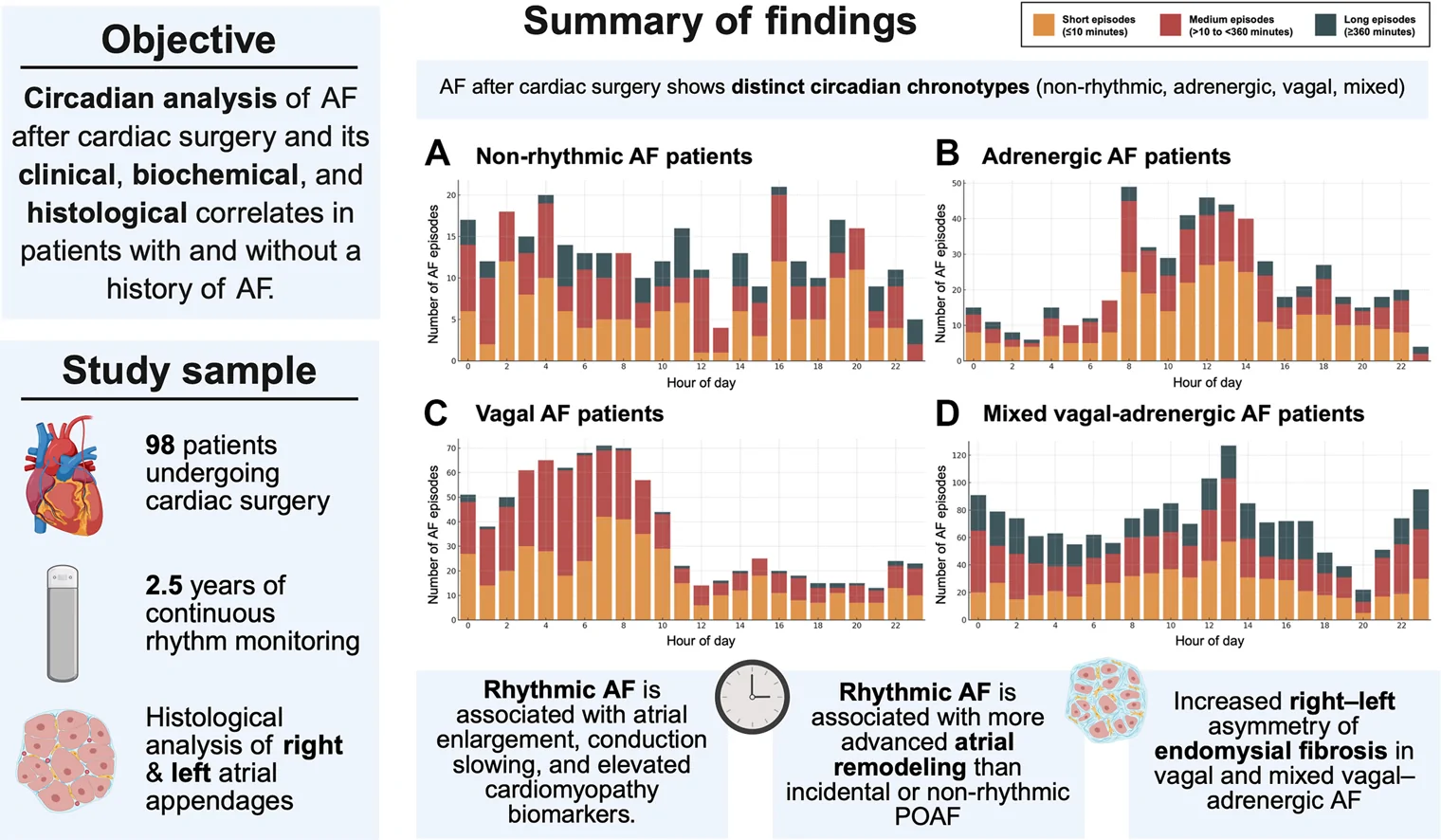
Circadian analysis of postoperative atrial fibrillation (POAF) in 98 cardiac surgery patients, using 2.5 years of continuous rhythm monitoring and right/left atrial appendage histology. AF episodes clustered into four circadian chronotypes: non-rhythmic, adrenergic, vagal, and mixed vagal-adrenergic. Rhythmic AF (adrenergic, vagal, or mixed) was linked to atrial enlargement, conduction slowing, elevated cardiomyopathy biomarkers, and greater atrial remodeling than non-rhythmic POAF, with increased right-left endomysial fibrosis asymmetry in the vagal and mixed groups.

**Key Findings**

▪ Atrial fibrillation (AF) after cardiac surgery, captured by 2.5 years of continuous monitoring in 98 patients, occurred in 54.1% and exhibited markedly different circadian behavior depending on preoperative rhythm history. AF recurrence in patients with prior AF showed a pronounced morning onset peak (08:40 AM), whereas new-onset postoperative AF in patients without prior AF was uniformly distributed across the 24-hour cycle.
▪ Of 26 patients with sustained postoperative AF (≥20 episodes), the majority (20/26) exhibited rhythmic circadian behavior that clustered into 3 distinct chronotypes, adrenergic (n = 9), vagal (n = 7), and mixed vagal-adrenergic (n = 4), whereas 6 were non-rhythmic, indicating that sustained AF after cardiac surgery follows individualized chronobiological trajectories rather than random onset dynamics.
▪ Rhythmic AF, and most prominently the mixed vagal-adrenergic chronotype, was associated with more advanced atrial remodeling (larger atrial volumes, prolonged PR intervals, higher BMP10 levels, and greater right-atrial endomysial fibrosis with pronounced right-to-left fibrosis asymmetry), suggesting that the structural substrate underlies not only AF susceptibility but also the persistence and rhythmic expression of AF after cardiac surgery.

## Introduction

New-onset early postoperative atrial fibrillation (early-POAF) is a frequent complication after cardiac surgery, occurring in up to 40% of patients, depending on the type of surgery performed.^1^ It is hypothesized that early-POAF occurs as a consequence of a combination of an ingrained preoperative arrhythmogenic substrate and perioperative triggers such as inflammation, oxidative stress, and sympathetic activation.^1^^,^^2^ However, a substantial number of patients who have experienced early-POAF will develop post-discharge late-POAF recurrences, even after the surgical triggers have disappeared.^3^^,^^4^ Similarly, in patients with a preoperative AF history, early AF recurrences after surgical AF ablation appear to be strongly associated with late AF recurrences.^5^

Although surgery-associated triggers are likely responsible for initiation of AF episodes during the early postoperative phase, the potential triggers for late AF episodes after cardiac surgery remain unknown. In non-surgical AF patients, the autonomic nervous system (ANS) was previously recognized as an important modulator of atrial cellular electrophysiology and a possible contributor to the arrhythmogenic substrate promoting AF initiation.^6^ Based on the fluctuation in ANS activity, different types of AF phenotypes were proposed, with patients experiencing mostly daytime AF episodes being considered adrenergic-mediated, and patients with nighttime AF episodes as vagal-mediated AF.^7^ Although these variations in AF occurrence throughout the day, also called circadian patterns, are partially attributed to the activity of the ANS, other mechanisms which are closely related and mediated by the ANS, such as central and cardiomyocyte-specific circadian clocks, potentially play a major role in AF initiation.^8^, ^9^, ^10^

Previous studies have described the circadian variations in AF occurrence in non-surgical patients; however, there is currently no data describing the circadian patterns of AF after cardiac surgery.^11^, ^12^, ^13^ Therefore, the main objective of the current study is to determine the circadian patterns of AF after cardiac surgery and to identify the clinical, biochemical, and histological characteristics of different circadian chronotypes in patients with and without a preoperative AF history during 2.5 years of continuous rhythm monitoring.

## Methods

### Study sample

After approval of the study protocol by the local ethical committee, consecutive patients were prospectively included in the RACE V–Tissue Bank Project at the Maastricht University Medical Centre (clinicaltrials.gov identifier NCT03124576). Written informed consent was obtained from all patients before inclusion in the study. Eligible patients were 18 years of age or older, underwent first cardiac surgery, and had a life expectancy of >2.5 years. Subjects with a history of AF, as well as those without a history of AF, were included. Patients undergoing standalone surgical AF ablation, had undergone prior cardiac surgeries, or who had congenital heart disease were excluded. Objectives, designs, and study procedures have been published previously.^14^ The research reported in this paper adhered to the Declaration of Helsinki and was approved by the local Medical Ethics Committee of Maastricht University Medical Centre.

### Pathophysiological assessment

Preoperatively, all patients underwent transthoracic echocardiography (TTE), standard 12-lead electrocardiography (ECG), and blood biomarker assessment according to a prespecified protocol.^14^^,^^15^ TTE was performed within 6 months before cardiac surgery. Left ventricular ejection fraction was calculated using the biplane Simpson method. Measurements of left atrial (LA) volumes were performed in the 4- and 2-chamber views using the biplane area-length method. Right atrial (RA) volumes were calculated in the 4-chamber view. Standard 12-lead ECGs were obtained for all patients at least 3 months before surgery. All measurements were performed in MUSE^TM^ (GE Medical Systems Information Technologies, Inc., Milwaukee, WI). Preoperative venous blood samples were drawn 1 day before cardiac surgery. For all patients, preoperative data on clinical history, comorbidity profile, and clinical presentation were gathered from medical records. Also, data regarding postoperative course in the intensive-care unit and surgical ward were extracted and analyzed.

### AF monitoring and definitions

Continuous rhythm monitoring in the RACE V–Tissue Bank Study has been described previously.^14^^,^^15^ In short, all patients received an implantable loop recorder (ILR) (Reveal Linq™, Medtronic, Minneapolis, MN, USA) and were continuously monitored for 2.5 years. AF episodes detected as text files only were excluded due to a false-positive rate of up to 80%.^16^ Episodes were included only if recorded on an ECG trace by the ILR and confirmed by the investigators. Early AF was defined as AF episodes occurring within the first 30 postoperative days, and late AF as episodes beyond this period.^17^^,^^18^ The 30-day threshold for classifying late AF episodes was chosen to exclude surgery-related triggers expected to subside after early recovery. To distinguish mechanistically distinct populations, the term “POAF” is used for AF episodes occurring in patients without a preoperative AF history (ie, new-onset postoperative AF), whereas postoperative AF in patients with a preoperative AF history is referred to as “postoperative AF recurrence;” both populations are reported separately wherever this distinction is meaningful. AF burden was defined as the percentage of time spent in AF. The longest and median episode durations, median ventricular rate (V-rate), and total number of AF episodes were calculated for 3 postoperative phases: early, late, and overall (entire follow-up). For analyses of pattern timing across the 24-hour cycle, circadian periods were defined a priori as night (00:00–05:59), morning (06:00–11:59), afternoon (12:00–17:59), and evening (18:00–23:59).

### Circadian chronotyping analysis

To characterize individual patterns of AF susceptibility and temporal behavior, patients with ≥20 confirmed AF episodes after surgery were included in a chronotyping analysis encompassing circadian onset, phase evolution, and longitudinal AF-burden progression patterns. This episode threshold was set at ≥20 to balance inclusion breadth with the statistical robustness required for circular statistics, time-series smoothing, and trajectory modeling.^19^ Patients with fewer episodes were excluded and labeled as having incidental AF, as they provided insufficient data to identify non-random circadian clustering or meaningful temporal trends. Patients were included in the chronotyping analysis regardless of preoperative rhythm history.

Circadian chronotype analysis was performed over the full 2.5-year follow-up to determine each patient’s preferred time of AF onset and to examine whether episode characteristics varied by circadian phase. Episode onset times were converted to circular angles (0–2π) based on minutes since midnight. The Rayleigh test was used to assess deviation from a uniform distribution, with the mean resultant length (R) quantifying phase concentration and the circular mean representing the preferred onset time. Patients were classified as non-rhythmic (Rayleigh *P* ≥ .05 and R < 0.20), rhythmic unimodal (Rayleigh *P* < .05 and R ≥ 0.20, single onset peak), or rhythmic multimodal (Rayleigh *P* < .05 and R < 0.20, multiple onset peaks). Episode duration and V-rate were plotted against onset time and smoothed using local regression (LOWESS, bandwidth = 0.2) to visualize circadian trends and identify vagal-dominant (nocturnal, long, slow), adrenergic-dominant (diurnal, short, fast), or mixed vagal–adrenergic (multimodal onset pattern) chronotypes. Based on the combined results of onset timing, duration, and V-rate analyses, patients were classified as: (1) adrenergic chronotype (rhythmic unimodal, predominantly diurnal, shorter episodes with higher V-rate), (2) vagal chronotype (rhythmic unimodal, predominantly nocturnal, longer episodes with low V-rate), and (3) mixed vagal–adrenergic chronotype (rhythmic multimodal, showing both diurnal shorter, high V-rate episodes and nocturnal longer, low V-rate episodes).

Next, circadian phase evolution was evaluated using a hybrid time-windowing approach, with a single window covering the first 3 postoperative months, followed by consecutive 6-month windows during late follow-up to assess changes in the preferential AF chronotype. For each window, the circular mean onset time and Rayleigh *P*-value were calculated to classify patients as having a stable rhythm (sustained, significant preferential onset time with consistent peak and unchanged chronotype classification) or a drifting rhythm (gradual phase shift, change in preferential onset peak, or shift in chronotype classification over time).

Lastly, AF burden trajectories were analyzed using the same time windows as the circadian phase evolution analysis. Within each window, the total AF episode duration was divided by the total number of minutes in that window, yielding a burden percentage over time. Patients were then categorized as having stable, increasing, or decreasing burden profiles.

### Adverse events during the follow-up

During the follow-up-period, a broad range of clinical adverse events was measured, which included stroke (hemorrhagic and ischemic), systemic embolism, vascular interventions, major bleeding, myocardial infarction, heart failure requiring hospitalization, electrical cardioversions, emergency room (ER) visits because of arrhythmia, ER visits with any cause, pulmonary vein ablation, and the need for pacemaker or cardioverter-defibrillator implantation. Additionally, “any event” (combination of all adverse events) and “any cardiovascular event” (combination of all adverse events excluding ER visits with any cause) were determined as 2 composite parameters.

### Histological analysis

Snap-frozen tissue samples of the LA and RA appendage (LAA/RAA) were collected during cardiac surgery and analyzed as previously described.^20^^,^^21^ In brief, all samples were cryosectioned (6 μm) and stained with wheat germ agglutinin (WGA) (connective tissue), mouse monoclonal anti-CD31 (capillary density), and Rabbit monoclonal anti-vimentin (fibroblast density). Next, slides were submerged in ice-cold acetone for 10 minutes to fixate antigens. Non-specific binding sites were blocked by application of a blocking solution (2 m/v% fraction V BSA and 0.3 M glycine) for 60 minutes. WGA conjugated to Alexa 594 (1:200; ThermoFisher, Netherlands) was applied for 120 minutes, and slides were mounted with ProLong Gold Antifade (ThermoFisher, Netherlands). Images of non-overlapping, transversely cut myocytes were captured at 400× magnification using a Leica DM4B microscope with an MC170 HD camera. Automated morphometric analysis was performed with JavaCyte.^20^ The fraction of WGA-positive pixels quantified extracellular matrix (fibrosis). Cardiomyocyte size was expressed as minimal Feret diameter, and inter-cardiomyocyte distances represented endomysial fibrosis.^20^ Capillary and fibroblast densities were calculated as the number of CD31- and vimentin-positive cells per myocyte, respectively.^20^

### Statistical analysis

All continuous variables were assessed for normality using the Shapiro–Wilk test. Data are presented as mean ± standard deviation or as median with interquartile range (IQR), depending on the distribution. Categorical variables are presented as absolute counts with corresponding proportions (%). Univariate group differences were evaluated using a one-way analysis of variance, Kruskal–Wallis test, or χ^2^ test, as appropriate for data type, distribution, and variance. For post-hoc comparisons, Bonferroni correction was applied.

The 24-hour onset and termination times of AF episodes were converted to angular values (degrees, 0°–360°) for circular analysis. Circadian AF onset patterns were assessed using the Rayleigh test to evaluate deviation from a uniform circular distribution, with the null hypothesis assuming isotropy (no preferred direction).

A *P*-value of < .05 was considered statistically significant. Given the exploratory nature of the study, rather than formal hypothesis testing, no additional correction for multiple testing was applied beyond post-hoc comparisons. All statistical analyses were performed in “RStudio Version 1.2.1335” (*circular* package) and Statistical Package for the Social Sciences version 26.0 (IBM Corp., Armonk, NY) software for statistical analysis.^22^

## Results

### Patient characteristics

A total of 98 consecutive patients who completed 2.5 years of continuous rhythm monitoring were included. The median age was 68 years (IQR, 63–72), and the majority of patients were male (76.5%) (Table 1). Most patients underwent coronary artery bypass grafting (68.4%) as the primary indication for surgery, and 72.4% (n = 71) had no history of preoperative AF. Among the 27 patients with a history of AF (27.6%), 16 had paroxysmal AF (16.3%) and 11 had persistent AF (11.2%). The majority of patients with preoperative AF (85.2%) underwent concomitant surgical ablation, which was defined as surgical ablation performed during the index cardiac procedure, including pulmonary vein isolation (25.9%), left-sided Maze (44.5%), or full Cox Maze IV (14.8%).

**Table 1:** Patient characteristics

Variable	Overall cohort (n = 98)	No AF-history (n = 71)	AF-history (n = 27)	*P*-value
*General patient characteristics*				
Age, years (IQR)	68 (63–72)	68 (60–72)	70 (67–72)	.116
Male sex (%)	75 (76.5)	57 (80.3)	18 (66.7)	.155
BMI, kg/m^2^ (IQR)	26.9 (24.7–29.3)	27.1 (24.7–29.1)	26.5 (25.0–29.6)	.803
NYHA classification	2 (1–2)	2 (1–3)	2 (2–3)	**.009**
Hypertension (%)	90 (91.8)	67 (94.4)	23 (85.2)	.138
Dyslipidemia (%)	85 (86.7)	64 (90.1)	21 (77.8)	.107
Diabetes mellitus (%)	18 (18.4)	13 (18.3)	5 (18.5)	.981
COPD (%)	13 (13.3)	10 (14.1)	3 (11.1)	.698
Pulmonary hypertension (%)	13 (13.3)	6 (8.5)	7 (25.9)	**.023**
Peripheral artery disease (%)	20 (20.4)	16 (22.5)	4 (14.8)	.397
Creatinine, μmol/L (IQR)	72.9 (61.8–83.1)	76.4 (64.3–85.5)	65.7 (56.7–75.7)	.146
Sleep apnea (%)	6 (6.1)	5 (7.0)	1 (3.7)	.538
Smoking history (%)	60 (61.2)	46 (64.8)	14 (51.9)	.240
Alcohol use (%)	59 (60.2)	43 (60.6)	16 (59.3)	.906
CHA_2_DS_2_-VASc-score (IQR)	3 (3–4)	3 (3–4)	3 (3–4)	.178
*Preoperative transthoracic echocardiography*				
LVEF, % (IQR)	57.0 (51.0–60.0)	56.0 (50.0–60.0)	58.5 (52.0–63.0)	.422
LAVImax, mL/m^2^ (IQR)	36.3 (27.6–45.9)	31.9 (26.4–40.8)	42.4 (36.7–62.2)	**<.001**
LAVImin, mL/m^2^ (IQR)	15.3 (10.4–28.1)	12.9 (9.2–19.3)	29.0 (19.2–43.5)	**<.001**
LATEF, % (IQR)	55.6 (42.7–62.4)	58.2 (52.5–65.1)	37.9 (30.4–47.4)	**<.001**
RAVImax, mL/m^2^ (IQR)	27.1 (21.0–35.1)	24.9 (18.7–32.9)	33.7 (27.9–37.8)	**<.001**
RAVImin, mL/m^2^ (IQR)	13.4 (9.0–17.9)	11.3 (8.5–14.9)	17.3 (15.6–23.2)	**<.001**
RATEF, % (IQR)	50.5 (39.3–57.0)	53.0 (44.5–59.8)	44.2 (22.9–53.5)	**<.001**
LVM, grams (IQR)	196.0 (159.0–242.0)	188.0 (150.0–241.5)	213.5 (164.0–267.0)	.298
LVMI, grams/m^2^ (IQR)	95.7 (81.1–127.9)	94.3 (79.9–126.7)	100.3 (84.4–131.3)	.243
E-wave, cm/s (IQR)	74 (61–93)	69 (61–83)	94 (61–140)	**.002**
*Preoperative 12-lead electrocardiography*				
PR-interval, msec (IQR)	163 (148–184)	160 (148–182)	176 (160–188)	.164
P-wave duration, msec (IQR)	116 (108–124)	112 (104–124)	124 (116–136)	**.011**
QRS-duration, msec (IQR)	96 (86–104)	96 (85–104)	98 (86–106)	.855
QT-interval, msec (IQR)	400 (380–428)	404 (384–426)	388 (367–429)	.193
QTc-interval, msec (IQR)	423 (408–447)	421 (409–442)	434 (409–455)	.267
*Preoperative biomarkers*				
ANG-2, ng/mL (IQR)	2.07 (1.60–2.90)	1.89 (1.52–2.33)	3.15 (2.15–5.40)	**<.001**
BMP-10, ng/mL (IQR)	1.84 (1.66–2.13)	1.73 (1.61–1.96)	2.27 (1.92–2.60)	**<.001**
DKK-3, ng/mL (IQR)	54.4 (47.9–62.1)	53.1 (47.5–60.2)	59.6 (52.6–66.1)	**.026**
ESM-1, pg/mL (IQR)	1817.3 (1533.3–2409.3)	1755.5 (1520.4–2057.5)	2641.6 (1961.9–3123.5)	**.001**
FGF-23, ng/mL (IQR)	154.2 (120.8–201.1)	139.8 (109.6–160.6)	180.0 (155.6–217.9)	**.011**
IGFBP-7, ng/mL (IQR)	102.2 (89.8–114.5)	97.4 (87.9–109.1)	114.5 (104.5–125.4)	**.039**
NT-proBNP, pg/mL (IQR)	1222.3 (537.8–2838.5)	838.2 (475.1–1763.3)	3297.5 (1498.2–4315.9)	**<.001**
GDF-15, pg/mL (IQR)	1352.0 (997.1–2143.0)	1297.3 (978.2–1949.8)	1393.0 (1028.0–2859.5)	.442
IL-6, pg/mL (IQR)	3.45 (2.11–6.64)	3.29 (2.35–5.85)	4.66 (1.82–7.37)	.656
Pro-BNP2, pg/mL (IQR)	270.5 (112.6–840.8)	175.0 (93.5–532.6)	1008.1 (277.8–1665.5)	**<.001**
Troponin T, pg/mL (IQR)	13.9 (10.3–18.9)	14.1 (10.7–18.2)	13.3 (9.7–19.0)	.255
*Surgical data*				
Type of operation (%)	98 (100)	71 (100)	27 (100)	**<.001**
CABG (%)	67 (68.4)	56 (78.9)	11 (40.7)	
AVR (%)	13 (13.2)	9 (12.7)	4 (14.8)	
MV surgery (%)	18 (18.4)	6 (8.4)	12 (44.5)	
Rhythm surgery (%)	98 (100)	71 (100)	27 (100)	*NA*
No rhythm surgery (%)	75 (76.5)	71 (100)	4 (14.8)	
PVI (%)	7 (7.1)	0	7 (25.9)	
Left Maze (%)	12 (12.2)	0	12 (44.5)	
Full Cox Maze (%)	4 (4.1)	0	4 (14.8)	
*Postoperative data*				
ICU admission, days (IQR)	1 (1–2)	1 (1–2)	1 (1–2)	.373
Hospitalization, days (IQR)	6 (5–8)	6 (5–8)	7 (6–10)	.092
Diuretics at discharge (%)	43 (43.9)	24 (33.8)	19 (70.4)	**.001**
Beta blocker at discharge (%)	88 (89.8)	67 (94.4)	21 (77.8)	**.015**
Anticoagulation at discharge (%)	43 (45.3)	18 (26.1)	25 (96.2)	**<.001**
AAD at discharge (%)	9 (9.2)	4 (5.6)	5 (18.5)	**.048**
Sotalol at discharge (%)	8 (8.2)	4 (5.6)	4 (14.8)	.138
Amiodarone at discharge (%)	1 (1.0)	0 (0)	1 (3.7)	.103

Bold values indicated significance at alpha threshold of 0.05.

ANG-2 = angiopoietin 2; AVR = aortic valve replacement; BMI = body mass index; BMP-10 = bone morphogenic protein 10; CABG = coronary artery bypass grafting; COPD = chronic obstructive pulmonary disease; DKK-3 = dickkopf 3; ESM-1 = endothelial cell-specific molecule 1; FGF-23 = fibroblast growth factor 23; GDF-15 = growth differentiation factor 15; ICU = intensive care unit; IGFBP-7 = insulin-like growth factor-binding protein 7; IL-6 = interleukin 6; IQR = interquartile range; LATEF = left atrial total ejection fraction; LAVI = left atrial volume indexed for body surface area; LVEF = left ventricle ejection fraction; LVMI = left ventricular mass indexed for body surface area; MV = mitral valve; ng = nanogram; NT-proBNP = n-terminal brain natriuretic peptide; NYHA = New York Heart Association; pg = picogram; pro-BNP2 = pro brain natriuretic peptide 2; PVI = pulmonary vein isolation; RATEF = right atrial total ejection fraction; RAVI = right atrial volume indexed for body surface area; SR = sinus rhythm.

### Circadian differences between rhythm groups

AF after cardiac surgery occurred in 53 patients (54.1%), totaling 3,607 episodes (Table 2). Because clinical and arrhythmic characteristics did not differ between paroxysmal and persistent AF rhythm histories (Table 1, Table 2), these categories were analyzed as a single subgroup (AF-history). Overall, AF after cardiac surgery exhibited a non-uniform circadian distribution, with a peak onset at 08:40 AM (95% confidence interval [CI], 07:57–09:22; *P* < .001) (Figure 1A). A similar pattern was observed among patients with preoperative AF history (mean onset time 08:40 AM; 95% CI, 07:57–09:22; *P* < .001) (Figure 1B). In contrast, patients without a preoperative AF history exhibited a uniform distribution, indicating no preferential circadian occurrence (Figure 1C). Compared with patients without preoperative AF history, those with preoperative AF had more AF episodes in the morning (32.5% vs 21.5%, *P* < .001) and fewer in the evening (14.6% vs 25.4%, *P* < .001). Similar trends were seen for late AF recurrences (Table 2).

**Table 2:** Incidence and occurrence patterns of atrial fibrillation

Variable	Overall cohort (n = 98)	No AF history (n = 71)	AF History (n = 27)	*P*-value
*AF incidence and patterns*				
Any AF (%)	53 (54.1)	29 (40.8)	24 (88.9)	**<.001**
AF-episodes per patient (IQR)	19 (4–52)	8 (3–29)	42 (9–70)	**.018**
AF-episode ventricular rate (IQR)	107 (95–125)	108 (98–125)	105 (94–128)	.775
AF-episode duration (%)	3607 (100)	965 (100)	2642 (100)	**<.001**
<10 min	1662 (46.1)	517 (53.6)	1145 (43.4)	
10 min to 6 h	1302 (36.1)	350 (36.3)	952 (36.0)	
>6 h	643 (17.8)	98 (10.2)	545 (20.6)	
AF-episode circadian occurrence (%)	3607 (100)	965 (100)	2642 (100)	**<.001**
Night	956 (26.5)	244 (25.3)	712 (26.9)	
Morning	1065 (29.5)	207 (21.5)	858 (32.5)	
Afternoon	957 (26.5)	269 (27.9)	688 (26.0)	
Evening	629 (17.4)	245 (25.4)	384 (14.6)	
*Early AF incidence and patterns*				
Early AF first 30 d (%)	48 (49.0)	27 (38.0)	21 (77.8)	**<.001**
Early AF first 30 d only (%)∗	14 (14.3)	8 (11.3)	6 (22.2)	.166
AF-episode per patient (IQR)	7 (2–24)	4 (2–14)	13 (3–38)	.187
AF-episode ventricular rate (IQR)	114 (98–131)	118 (102–133)	103 (82–125)	.146
AF-episode duration (%)	730 (100)	311 (100)	419 (100)	.245
<10 min	309 (42.3)	141 (45.3)	168 (40.1)	
10 min to 6 h	307 (42.1)	128 (41.2)	179 (42.7)	
>6 h	114 (15.6)	42 (13.5)	72 (17.2)	
AF-episode circadian occurrence (%)	730 (100)	311 (100)	419 (100)	**.004**
Night	216 (29.6)	92 (29.6)	124 (29.6)	
Morning	228 (31.2)	82 (26.4)	146 (34.8)	
Afternoon	157 (21.5)	65 (20.9)	92 (22.0)	
Evening	129 (17.7)	72 (23.2)	57 (13.6)	
*Late AF incidence and patterns*				
Late AF after first 30 d (%)	39 (39.8)	21 (29.6)	18 (66.7)	**<.001**
Late AF after first 30 d only (%)†	5 (5.1)	2 (2.8)	3 (11.1)	.095
AF-episode per patient (IQR)	16 (3–41)	4 (2–23)	23 (15–85)	**.002**
AF-episode ventricular rate (IQR)	100 (89–125)	98 (90–125)	102 (88–125)	.860
AF-episode duration (%)	2877 (100)	654 (100)	2223 (100)	**<.001**
<10 min	1353 (47.0)	376 (57.5)	977 (43.9)	
10 min to 6 h	995 (34.6)	222 (33.9)	773 (34.8)	
>6 h	529 (18.4)	56 (8.6)	473 (21.3)	
AF-episode circadian occurrence (%)	2877 (100)	654 (100)	2223 (100)	**<.001**
Night	740 (25.7)	152 (23.2)	588 (26.5)	
Morning	837 (29.1)	125 (19.1)	712 (32.0)	
Afternoon	800 (27.8)	204 (31.2)	596 (26.8)	
Evening	500 (17.4)	173 (26.5)	327 (14.7)	

Bold values indicated significance at alpha threshold of 0.05.

AF = atrial fibrillation; IQR = interquartile range.

Night = 00:00–05:59; morning = 06:00–11:59; afternoon = 12:00–17:59; evening = 18:00–23:59.

∗ Patient who only had AF within the first 30 days after surgery.

† Patient who only had AF after the first 30 days after surgery.

**Figure 1:**
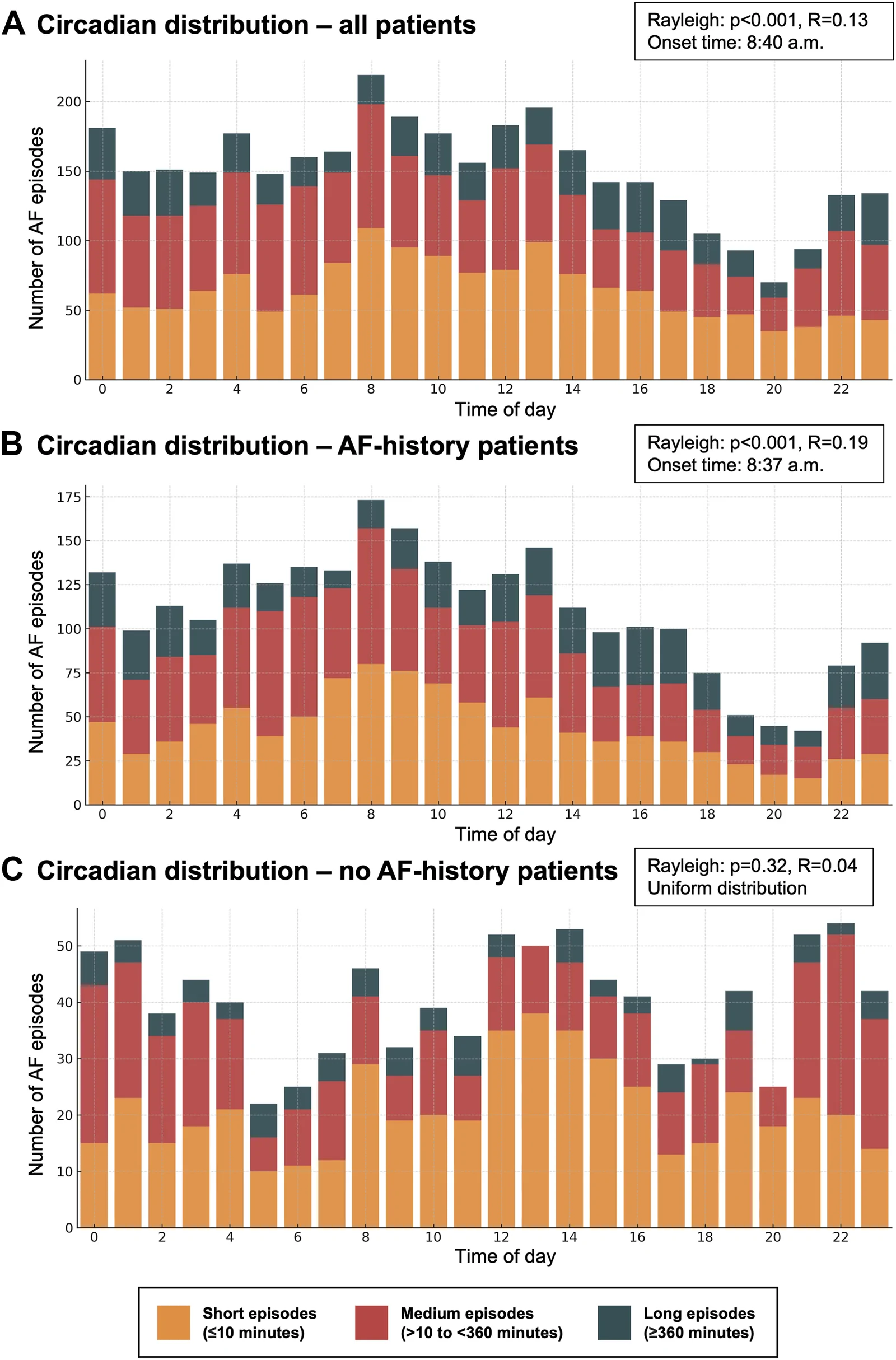
Distribution of postoperative AF episodes according to rhythm history. **A:** Circadian distribution of AF episodes during the follow-up in all patients. **B:** Circadian distribution of AF episodes during the follow-up in patients wih history of AF. **C:** Circadian distribution of AF episodes during the follow-up in patients without any history of AF. AF = atrial fibrillation.

In addition to group-level findings, patient-level AF incidence and circadian distribution are illustrated in Figure 2A–B. Figure 2C presents 3 representative patients with distinct rhythm histories, each demonstrating progressive AF dynamics over time, characterized by episode clustering during the night and early morning hours.

**Figure 2:**
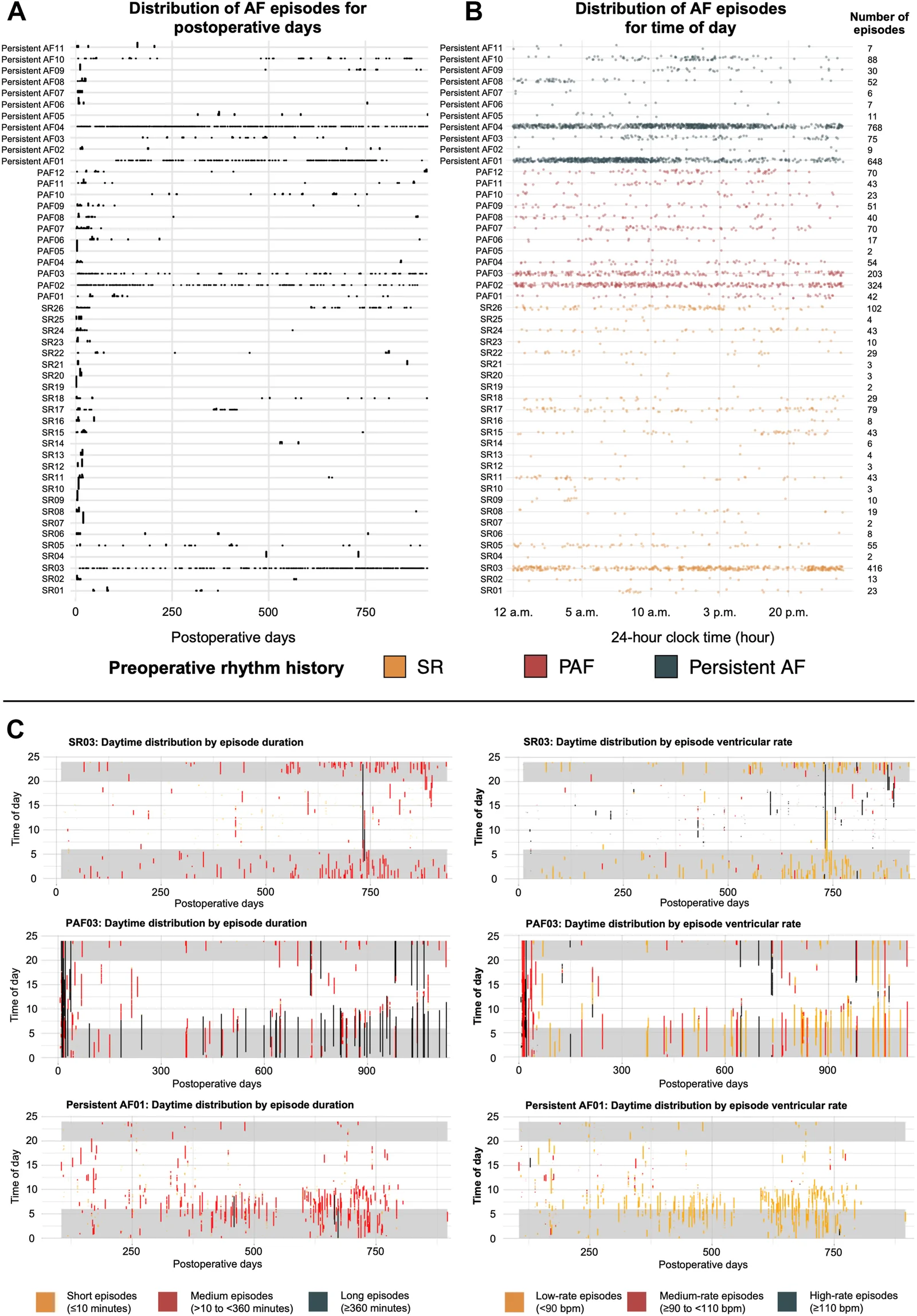
Distribution of AF episodes after cardiac surgery. **A:** Distribution of AF episodes during the follow-up. **B:** Circadian distribution of AF episodes. **C:** Circadian distribution of AF episodes for 3 subjects with different preoperative rhythm histories. AF = atrial fibrillation; PAF = paroxysmal atrial fibrillation; SR = sinus rhythm.

### Circadian chronotype analysis

#### Classification

Among the 53 patients with AF after cardiac surgery, 27 had incidental episodes (<20 total; median 4, IQR, 2–9), while 26 experienced sustained AF (≥20 episodes; median 53, IQR, 42–88) and were included in the circadian chronotype analysis (Figure 3). Of these, 20 exhibited a rhythmic episode pattern and 6 were non-rhythmic (Figure 4A). Within the rhythmic group, 9 patients had an adrenergic chronotype, marked by daytime episodes peaking in the afternoon (Figure 4B), and 7 had a vagal chronotype, with episodes clustering at night or early morning (Figure 4C). 4 patients showed a mixed vagal–adrenergic chronotype, characterized by a bimodal distribution reflecting both daytime and nighttime activity patterns (Figure 4D).

**Figure 3:**
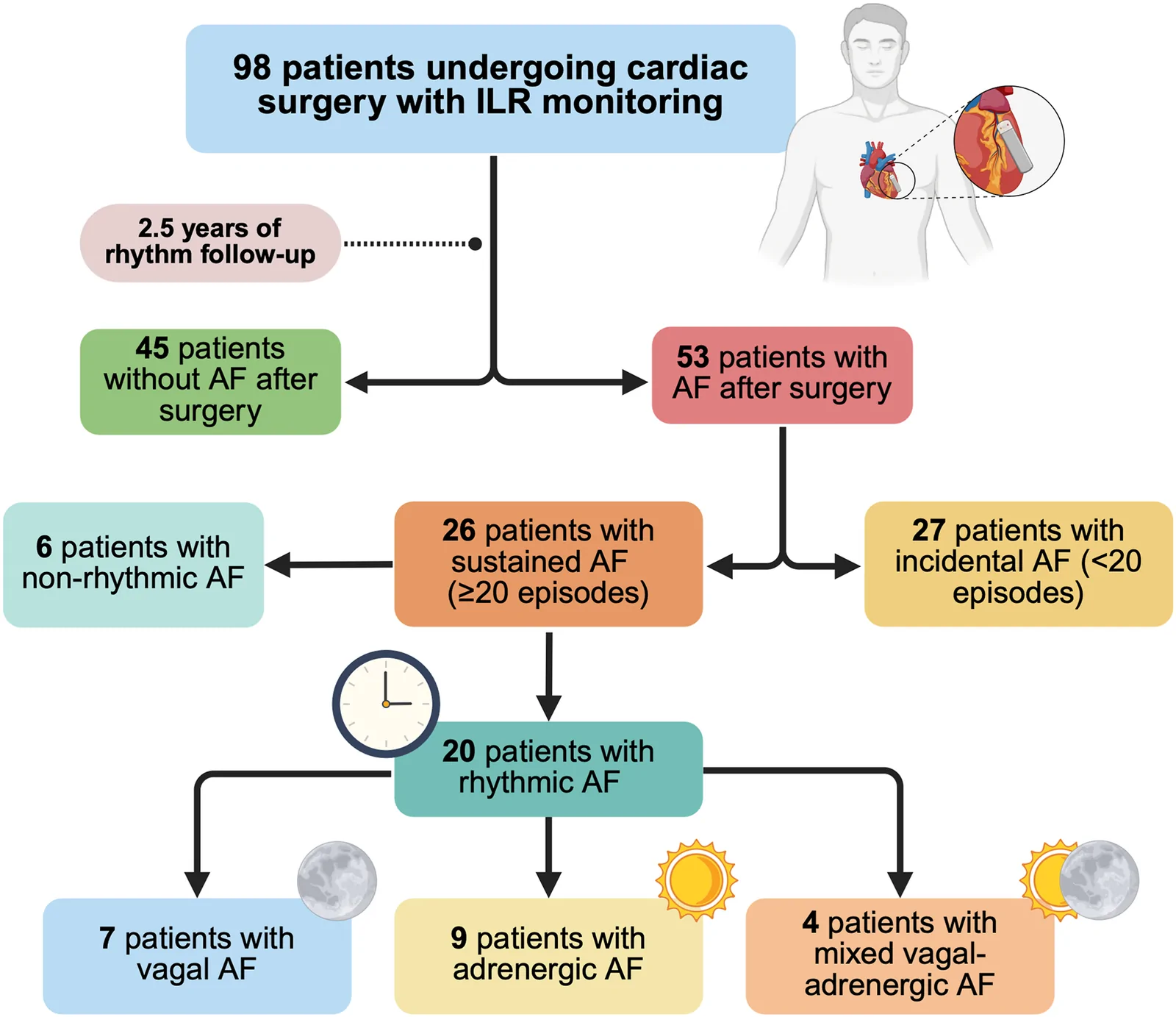
Circadian chronotype distribution diagram. ILR = implantable loop recorder.

**Figure 4:**
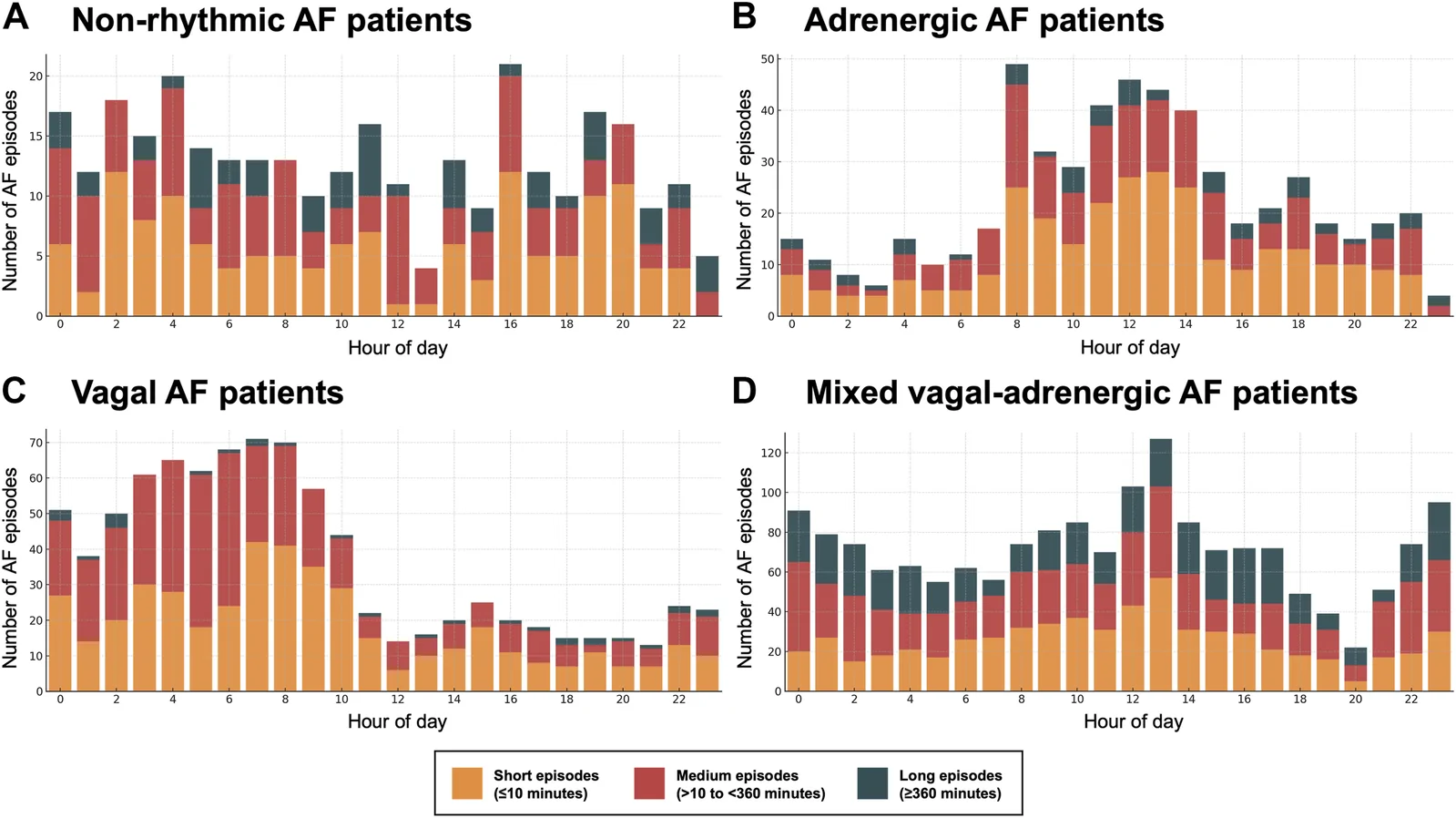
Distribution of AF episodes according to circadian chronotype. **A:** Circadian distribution of AF episodes in non-rhythmic patients. **B:** Circadian distribution of AF episodes in adrenergic patients. **C:** Circadian distribution of AF episodes in patients with vagal chronotype. **D:** Circadian distribution of AF episodes in patients with vagal–adrenergic chronotype. AF = atrial fibrillation.

#### AF characteristics

Compared with patients who developed incidental AF, those with non-rhythmic or rhythmic AF had a higher overall AF burden (*P* < .001), lower AF density (*P* < .001), and a higher overall number of AF episodes (*P* < .001) (Supplemental Table 1). The rhythmic subgroup was further characterized by a lower Vrate (*P* < .001) and longer maximum episode duration (*P* = .011) as compared with patients with incidental AF (Supplemental Table 1). Both rhythmic and non-rhythmic subgroups more frequently exhibited late-AF episodes (*P* = .009) with a higher late-phase burden (Supplemental Table 1). Within the rhythmic subgroup, patients with a mixed vagal–adrenergic chronotype experienced the highest episode counts overall (*P* = .023) and during the late phase (*P* = .038) as compared to the other chronotypes (Table 3 and Supplemental Table 2).

**Table 3:** Incidence and occurrence patterns of atrial fibrillation according to chronotype phenotypes

Variable	Incidental AF∗ (n = 27)	Non-Rhythmic AF(n = 6)	Adrenergic AF (n = 9)	Vagal AF (n = 7)	Mixed Vagal-Adrenergic (n = 4)	*P*-value
*AF incidence and patterns*						
AF burden, in % (IQR)	0.05 (0.01–0.16)	0.34 (0.14–4.40)	0.96 (0.16–2.55)	0.77 (0.13–1.96)	2.07 (1.24–70.3)	**<.001**
AF density (IQR)	0.98 (0.96–0.99)	0.72 (0.48–0.91)	0.85 (0.66–0.97)	0.91 (0.48–0.97)	0.64 (0.27–0.90)	**<.001**
AF-episode per patient (IQR)	4 (2–9)	52 (39–61)	70 (36–81)	42 (29–52)	370 (233–680)	**<.001**
AF-episode ventricular rate (IQR)	115 (100–138)	96 (84–128)	109 (98–116)	90 (70–118)	97 (94–104)	.068
Median episode duration per patient (IQR)	46 (4–220)	12 (8–4736)	8 (5–18)	12 (8–37)	34 (4–366)	.822
Longest episode duration per patient (IQR)	410 (34–1435)	1501 (804–11179)	2962 (866–5940)	856 (354–8181)	1747 (1309–11825)	.051
*Early AF incidence and patterns*						
Early AF first 30 d (%)	23 (85.2)	6 (100)	9 (100)	6 (85.7)	4 (100)	.543
Early AF burden, in % (IQR)	0.78 (0.01–3.63)	6.48 (2.83–42.5)	2.82 (0.62–5.11)	1.78 (0.001–7.35)	10.71 (1.48–30.4)	**.027**
AF-episode per patient (IQR)	4 (2–6)	31 (6–42)	13 (3–41)	19 (8–45)	38 (20–71)	**<.001**
AF-episode ventricular rate (IQR)	122 (107–146)	129 (100–133)	100 (82–107)	87 (61–107)	122 (98–123)	**.009**
Median episode duration per patient (IQR)	83 (12–367)	22 (14–148)	24 (4–570)	10 (6–398)	34 (5–76)	.054
Longest episode duration per patient (IQR)	259 (2–920)	846 (369–12799)	792 (249–926)	648 (0–856)	792 (136–1312)	.158
*Late AF incidence and patterns*						
Late AF after first 30 d (%)	15 (55.6)	6 (100)	8 (88.9)	6 (85.7)	4 (100)	**.045**
Late AF burden, in % (IQR)	0.003 (0.001–0.02)	0.10 (0.004–3.11)	0.04 (0.006–0.31)	0.31 (0.01–1.07)	1.75 (1.14–71.3)	**.013**
AF-episode per patient (IQR)	2 (1–5)	27 (8–42)	19 (10–69)	22 (12–192)	344 (195–615)	**<.001**
AF-episode ventricular rate (IQR)	111 (91–137)	92 (82–125)	109 (97–118)	92 (83–118)	97 (94–103)	.347
Median episode duration per patient (IQR)	6 (2–42)	7 (3–170)	6 (4–11)	20 (7–115)	34 (4–479)	.671
Longest episode duration per patient (IQR)	0 (0–26)	710 (18–8727)	68 (18–719)	316 (20–1084)	1328 (990–16755)	**<.001**

Bold values indicated significance at alpha threshold of 0.05. Duration of episodes is presented in minutes.

AF = atrial fibrillation; IQR = interquartile range.

∗ Defined as less than 20 episodes in total.

#### Clinical characteristics

Preoperative rhythm history significantly differed between AF groups (*P* < .001), with the patients with rhythmic AF having the highest prevalence of preoperative AF history (14/20, 70.0%) (Supplemental Table 3). Post-hoc analyses, however, did not reveal a significant difference between rhythmic and non-rhythmic AF groups (33.3% vs 70.0%, *P* = .105). The rhythmic AF group more frequently underwent valvular surgery (*P* = .013) and concomitant rhythm surgery (*P* < .001) compared with patients without AF, with incidental AF, or with a non-rhythmic AF pattern (Supplemental Table 3). Atrial remodeling features, including larger atrial volumes (LAVI in rhythmic AF: 40.4 mL/m^2^ [36.6–68.8] vs 30.0 mL/m^2^ [25.8–38.3] in patients without AF, *P* < .001), prolonged PR intervals (184 ms [166–210] vs 158 ms [146–178], *P* = .033), and elevated preoperative bone morphogenetic protein-10 levels (BMP10) (1.93 ng/mL [1.73–2.52] vs 1.71 ng/mL [1.59–1.94], *P* = .014), were most prominent in the rhythmic AF group, although they did not significantly differ from the other AF subgroups (Supplemental Table 3).

Comparison between the non-rhythmic and rhythmic chronotype subgroups, which were further divided into adrenergic, vagal, and mixed vagal–adrenergic patterns, showed that patients with a vagal AF pattern had significantly larger RA volume indexed for body surface area (RAVImax) (*P* = .049), particularly compared with those with an adrenergic pattern (Supplemental Table 4). Patients with mixed vagal–adrenergic pattern received rhythm control therapy after surgery (*P* = .040) more often (Supplemental Table 4).

#### Histological features

Overall, RAA samples were available in 80 patients, LAA samples in 74, and paired RAA–LAA samples in 63 patients. Patients with rhythmic AF had significantly larger LAA cardiomyocyte diameters and higher LAA capillary densities compared to those without AF or with incidental AF (*P* = .005 and *P* = .011, respectively) (Supplemental Table 5). Additionally, rhythmic AF was associated with increased RAA endomysial fibrosis relative to the no-AF, incidental AF, and nonrhythmic AF groups (*P* = .022) (Supplemental Table 5). Within the rhythmic group, patients with a mixed vagal–adrenergic chronotype had significantly higher RAA endomysial fibrosis levels than those with non-rhythmic or adrenergic chronotypes (*P* = .009), but not compared with vagal patients (Table 4, Supplemental Table 6). The individual variabilities between patients with different chronotypes are depicted in Figure 5A–C, where it is apparent that patients with adrenergic AF (Figure 5A) have less endomysial fibrosis as compared to the mixed vagal–adrenergic chronotype (Figure 5C).

**Table 4:** Histological characteristics of circadian chronotypes

Variable	No AF (n = 45)	Incidental AF∗ (n = 27)	Non-Rhythmic AF (n = 6)	Adrenergic AF (n = 9)	Vagal AF (n = 7)	Mixed Adrenergic-Vagal (n = 4)	*P*-value
*Left atrial characteristics*							
Myocyte diameter, in mm (IQR)	10.7 (10.1–11.6)	11.1 (10.9–11.8)	11.2 (10.1–11.5)	12.7 (11.3–13.8)	12.0 (11.2–12.6)	12.6 (10.2–17.4)	**.021**
EMF, in mm (IQR)	4.9 (4.3–5.6)	5.1 (4.6–5.5)	5.0 (4.5–5.5)	4.9 (4.6–6.3)	6.2 (5.2–7.0)	5.8 (4.7–6.9)	.409
ECM, in % (IQR)	23.2 (21.1–25.3)	20.7 (18.7–23.9)	24.2 (23.2–26.3)	18.5 (16.9–20.7)	22.4 (18.9–25.5)	24.7 (18.4–28.3)	.359
Capillary density, ratio (IQR)	0.23 (0.19–0.31)	0.20 (0.18–0.26)	0.21 (0.13–0.27)	0.42 (0.31–0.57)	0.30 (0.22–0.30)	0.38 (0.23–0.51)	**.027**
Capillary size, in mm (IQR)	4.21 (3.81–4.70)	4.47 (3.71–4.76)	4.54 (4.27–4.89)	4.06 (3.22–4.15)	4.92 (3.72–5.05)	4.23 (3.89–5.02)	.624
Fibroblast density, ratio (IQR)	0.47 (0.38–0.55)	0.48 (0.41–0.61)	0.43 (0.41–0.44)	0.62 (0.45–0.63)	0.58 (0.47–0.64)	0.70 (0.42–0.90)	.331
*Right atrial characteristics*							
Myocyte diameter, in mm (IQR)	10.8 (9.9–11.4)	11.5 (10.8–11.9)	10.9 (9.7–11.1)	11.2 (10.8–11.6)	11.7 (9.1–13.0)	11.5 (10.8–12.0)	.182
EMF, in mm (IQR)	6.0 (5.4–6.6)	6.3 (5.6–6.7)	5.9 (5.8–6.0)	6.4 (6.0–6.8)	6.8 (6.3–7.3)	9.6 (7.2–20.7)	**.019**
ECM, in % (IQR)	25.9 (22.9–27.9)	25.4 (21.5–28.4)	25.5 (24.0–26.6)	23.8 (21.4–25.4)	24.9 (23.0–27.6)	27.8 (24.8–30.2)	.769
Capillary density, ratio (IQR)	0.30 (0.25–0.35)	0.31 (0.26–0.32)	0.25 (0.22–0.37)	0.36 (0.29–0.59)	0.25 (0.15–0.45)	0.27 (0.24–0.28)	.842
Capillary size, in mm (IQR)	3.72 (3.22–4.05)	4.11 (3.61–4.38)	4.02 (3.89–4.92)	3.76 (3.00–4.49)	4.52 (3.39–4.58)	3.59 (3.45–3.84)	.504
Fibroblast density, ratio (IQR)	0.51 (0.40–0.58)	0.53 (0.43–0.65)	0.48 (0.45–0.66)	0.65 (0.45–1.05)	0.52 (0.50–0.75)	0.50 (0.46–0.53)	.870

Bold values indicated significance at alpha threshold of 0.05.

AF = atrial fibrillation; ECM = electromechanical coupling; EMF = electromechanical feedback; SD = standard deviation; mm = micrometer.

∗ Defined as less than 20 episodes in total.

**Figure 5:**
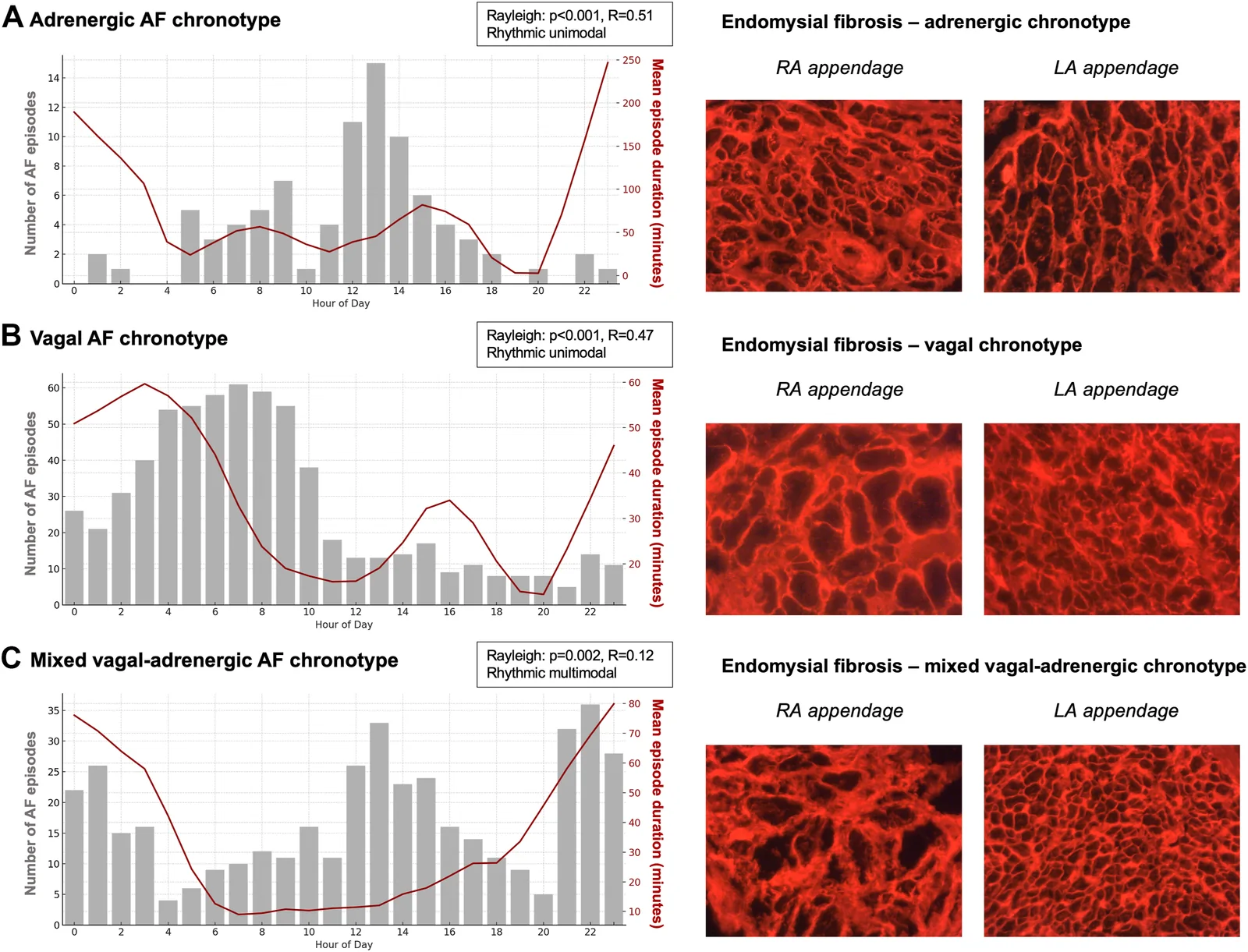
Distribution of AF episodes and histology according to circadian chronotype. **A:** Circadian distribution of AF episodes with corresponding histological findings in 1 patient with adrenergic AF. **B:** Circadian distribution of AF episodes with corresponding histological findings in 1 patient with vagal AF. **C:** Circadian distribution of AF episodes with corresponding histological findings in 1 patient with mixed vagal–adrenergic AF. LA = left atrium; RA = right atrium.

Paired analysis showed greater RAA–LAA fibrosis differences in mixed chronotype patients (*P* = .042), significantly exceeding those in non-rhythmic (*P* = .006), adrenergic (*P* = .038), and vagal (*P* = .044) groups (Supplemental Table 7).

#### AF progression patterns

Progression patterns were assessed in 26 patients with sustained AF (≥20 episodes), including 6 with non-rhythmic and 20 with rhythmic AF. None of the non-rhythmic patients showed a progressive phenotype, defined as an increase in AF burden (Figure 6A, Supplemental Table 8). Also, the Vrate during episodes remained stable in this group (Figure 6B, Supplemental Table 8). In contrast, patients with rhythmic AF demonstrated more variability, with 60% showing a decrease and 15% an increase in burden (Supplemental Table 8). However, these changes were not statistically different from the non-rhythmic group (*P* = .148), and ventricular rate fluctuations were also similar (*P* = .492) (Supplemental Table 8).

**Figure 6:**
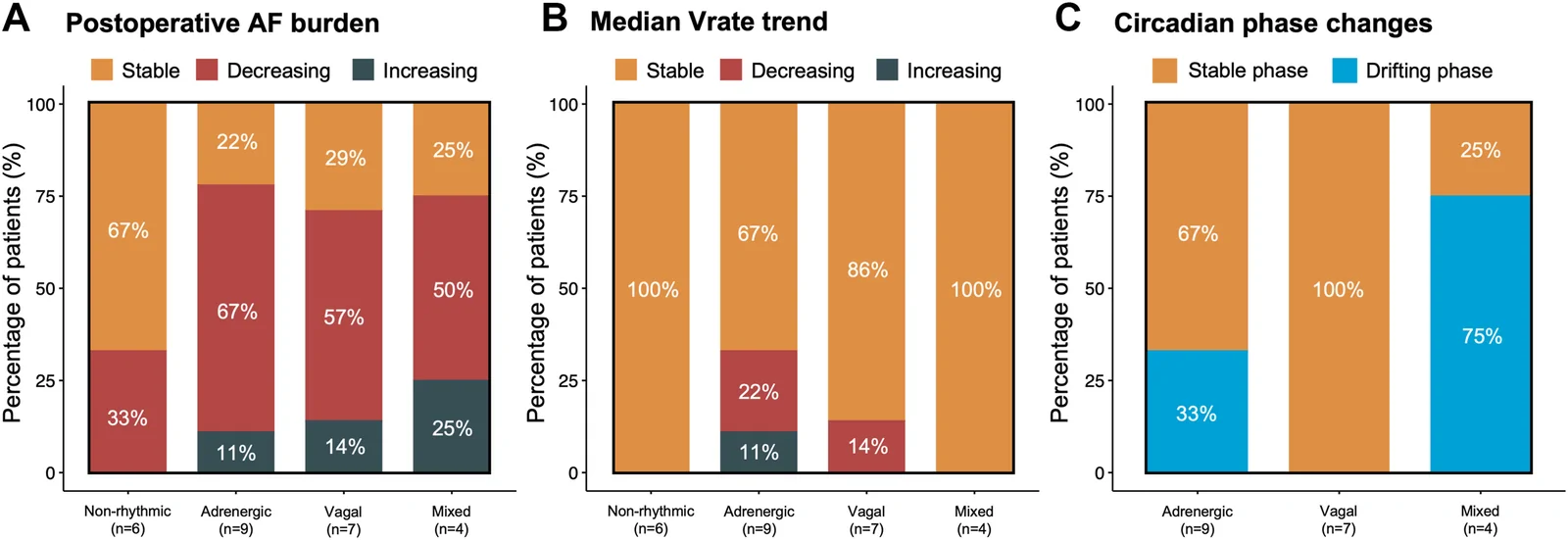
AF progression patterns according to circadian chronotypes. **A:** AF burden progression patterns according to circadian chronotypes. **B:** AF median ventricular rate progression patterns according to circadian chronotypes. **C:** AF circadian phase changes according to circadian chronotype. AF = atrial fibrillation; V-rate = ventricular rate.

Notably, patients with mixed vagal–adrenergic chronotypes more frequently exhibited circadian phase shifts than those with a vagal pattern (*P* = .007; Figure 6C, Supplemental Table 9). Patients with adrenergic AF had more shifts numerically (33.3 percent), but this difference was not significant (*P* = .090). Also, no significant differences in burden progression or rate variability were observed across AF chronotypes (Figure 6A–B and Supplemental Table 9).

#### Adverse events during follow-up

Patients with rhythmic AF presented more often with arrhythmias at the emergency department during follow-up compared with those without AF or with incidental AF (*P* < .001) (Supplemental Table 10). They also underwent electrical cardioversion more frequently (*P* = .002) and had more emergency visits for any cause (*P* = .033) (Supplemental Table 10). Interestingly, patients with an adrenergic AF chronotype accounted for the majority of arrhythmia-related emergency visits and presented to the emergency department more frequently than other AF chronotypes (*P* = .034) (Supplemental Table 11).

## Discussion

This study is among the few to explore circadian patterns of AF and the first to characterize AF chronotypes using long-term continuous rhythm monitoring after cardiac surgery. Assessing circadian arrhythmia dynamics in this setting is particularly relevant, as it enables identification of potentially distinct circadian patterns across different postoperative phases in which AF may be driven by different mechanisms. In this study, patients with a preoperative AF history displayed a pronounced morning peak in AF onset, whereas those with new-onset POAF exhibited a uniform 24-hour distribution. This divergence suggests that pre-existing atrial disease and altered autonomic regulation after surgery, possibly influenced by pulmonary vein ablation, may determine postoperative rhythm behavior. Specifically, increased adrenergic drive may contribute to POAF initiation, whereas surgical ablation may reduce vagally mediated AF as a result of autonomic denervation. Among patients with sustained POAF, most exhibited rhythmic episode patterns that clustered into adrenergic, vagal, and mixed vagal–adrenergic chronotypes. These chronotypes were associated with more advanced atrial remodeling, with the mixed vagal–adrenergic subtype showing the highest episode counts, most pronounced markers of atrial cardiomyopathy, and the greatest variability in RAA endomysial fibrosis. Rhythmic AF chronotype, particularly of the adrenergic type, was also linked to more frequent arrhythmia-related emergency visits and cardioversions, underscoring its clinical relevance. Collectively, these observations highlight that AF occurring after cardiac surgery is not a uniform postoperative entity but consists of distinct circadian subtypes reflecting differences in autonomic and structural substrates.

### Circadian patterns and AF progression

Several studies have investigated circadian patterns of AF occurrence in non-surgical populations, reporting varying preferential onset times ranging from predominantly daytime^11^^,^^23^^,^^24^ to nighttime^12^^,^^13^^,^^23^^,^^25^^,^^26^ AF patterns. However, most of these studies relied on the clinical presentation of AF or on intermittent ambulatory rhythm monitoring, which complicates accurate estimation of true circadian distribution given the high prevalence of asymptomatic episodes. Studies using ILR data to evaluate circadian AF patterns remain limited, but 2 recent reports identified distinct device-detected AF chronotypes, demonstrating both adrenergic and vagal onset profiles.^27^^,^^28^ The present findings suggest that the distinct circadian AF patterns observed after cardiac surgery likely reflect similar interindividual variations in chronotype. Sympathetic activation during early morning hours is a well-established trigger for AF onset, whereas enhanced vagal activity during rest and sleep promotes nocturnal or early-morning episodes.^29^ In the early postoperative phase, physiological autonomic oscillation is further disrupted by surgical stress, inflammation, and partial autonomic denervation, transiently altering the parasympathetic–sympathetic balance.^2^ In contrast, during the late postoperative phase, autonomic dysregulation and systemic inflammation largely subside, reducing immediate triggers for AF and allowing a return toward physiological equilibrium. This aligns with our observation of a predominantly stable or decreasing AF burden over time. The circadian chronotype established early after surgery generally persisted during follow-up, with only the mixed vagal–adrenergic group showing a notable phase shift. This subtype also exhibited the highest episode frequency and AF burden after surgery, although comparisons were limited by small sample size. Previous studies similarly associated the mixed vagal–adrenergic pattern with greater AF progression in non-surgical cohorts, possibly reflecting more advanced atrial cardiomyopathy in which arrhythmogenesis depends primarily on substrate rather than autonomic triggers.^27^ The underlying mechanisms, however, remain incompletely understood.

### Atrial remodeling and structural substrate

Beyond autonomic modulation, the observed AF chronotypes appear to be anchored in distinct structural substrates. A previous study reported that patients with a mixed vagal–adrenergic phenotype had more comorbidities and a higher risk of AF progression than those with predominantly nocturnal or daytime onset, suggesting that atrial remodeling may obscure intrinsic rhythmicity.^27^ Similarly, in the present cohort, rhythmic AF after cardiac surgery was associated with larger atrial volumes, prolonged PR intervals, and higher circulating BMP10 levels, all features indicative of advanced atrial remodeling.^30^ Histological analysis further demonstrated that patients with the rhythmic AF chronotype, particularly those with a mixed vagal–adrenergic chronotype, exhibited greater RAA endomysial fibrosis and pronounced right-to-left atrial fibrosis asymmetry. Taken together, these findings suggest that the degree and distribution of atrial remodeling, reflected by both clinical and histological characteristics, may determine not only AF susceptibility but also the persistence, stability, and rhythmic expression of AF over time. In this context, cardiac surgery itself, particularly valvular procedures involving atrial incisions, may unmask or precipitate the chronotype to which a patient is predisposed, since surgical scarring promotes a substrate-dominant, predominantly adrenergic phenotype consistent with patterns described in other conditions characterized by atrial remodeling, such as heart failure with preserved ejection fraction.^31^ In our cohort, the higher prevalence of valvular and concomitant rhythm surgery within the rhythmic AF group, particularly the adrenergic and mixed vagal–adrenergic chronotypes, is consistent with this concept.

Of note, most patients presenting with rhythmic AF chronotype in the current study underwent concomitant rhythm surgery, which may further influence autonomic regulation by disrupting both vagal and adrenergic atrial innervation and thereby attenuating rhythmicity during follow-up. Endocardial parasympathetic denervation has previously been shown to terminate vagally mediated AF, although this relationship has not been systematically investigated in patients undergoing surgical AF ablation.^32^ Interestingly, total cardiac denervation after heart transplantation has been associated with a markedly lower incidence of POAF compared with double-lung transplantation, in which pulmonary veins are reconnected without achieving complete cardiac autonomic denervation.^33^ To fully elucidate the impact of surgical AF ablation on individual circadian patterns, it is essential to establish the preoperative circadian rhythm and assess postoperative changes. Such insights may further clarify the contribution of autonomic regulation to AF initiation and persistence. Beyond surgical autonomic disruption, postoperative pharmacological therapy also modulates the autonomic substrate. The high background prevalence of beta-blocker therapy in our cohort (89.8% at discharge, with comparable rates across chronotypes) likely attenuates the absolute amplitude of adrenergically driven episodes, yet does not appear to prevent the classification of patients' preferential chronotype, since beta-blocker exposure was broadly similar across rhythmic subgroups. Antiarrhythmic drug use was relatively low (≤10% at discharge) and concentrated in patients with rhythmic AF, particularly those with concomitant surgical ablation; this is therefore unlikely to explain the observed chronotype differences but remains a residual confounder for the absolute episode burden.

### Clinical implications

Accurate identification of circadian AF chronotypes after cardiac surgery may be crucial for understanding the underlying pathophysiological mechanisms and for optimizing monitoring and therapeutic strategies. Although the present findings are primarily exploratory, they indicate that AF after cardiac surgery is a heterogeneous phenomenon, with patients exhibiting distinct preferential circadian chronotypes. In this cohort, rhythmic AF, particularly of the adrenergic type, was associated with higher rates of arrhythmia-related emergency visits and cardioversions. These observations suggest that AF occurrence patterns after cardiac surgery follow individualized chronobiological trajectories that may carry prognostic value for long-term rhythm outcomes. Although this requires confirmation in larger, prospective studies, recognizing such trajectories could refine postoperative monitoring and guide therapeutic intensity. For example, patients with adrenergic chronotypes may benefit from optimized rate control timed to periods of peak sympathetic activity, whereas those with vagal or mixed chronotypes may require broader rhythm control strategies. In the current era, the implementation of wearable devices and other intermittent rhythm monitoring methods could make circadian chronotype profiling more accessible, since continuous rhythm monitoring is not feasible for all patients. Such approaches may ultimately facilitate more personalized, chronotype-based treatment strategies for AF occurring after cardiac surgery, but also for patients with non-surgical AF.

### Limitations

The current study has several limitations that should be acknowledged. It was conducted at a single institution with a relatively small sample size, which may limit generalizability, particularly for subgroup analyses of circadian chronotypes. Although rhythm classification was based on continuous long-term monitoring, external factors, such as medication timing, sleep disturbances, and diurnal variations in activity, could have influenced arrhythmia onset patterns. In addition, varying surgical indications introduced heterogeneous mechanisms of atrial remodeling, and differences in surgical technique among surgeons may have affected outcomes. The influence of concomitant rhythm surgery, particularly pulmonary vein ablation, also warrants caution when interpreting findings related to autonomic modulation. Concomitant rhythm surgery may further have altered postoperative circadian patterns relative to the preoperative state, but this cannot be assessed in the present study because preoperative continuous rhythm monitoring was not performed. Importantly, the term “AF after cardiac surgery” is used in the current study to encompass both new-onset POAF and recurrent AF in patients with a preoperative AF history; while we report these populations separately wherever possible, a substantial proportion of late episodes (beyond 30 days), particularly in patients with prior AF, likely reflect the natural history of established AF rather than a direct, reversible consequence of surgery itself, and should be interpreted as such. Histological analyses were limited to atrial appendage tissue, which may not fully capture the extent of diffuse atrial remodeling. Furthermore, the observed associations between circadian phenotype, fibrosis, and clinical outcomes are observational in nature and cannot establish causality. Finally, because preoperative continuous rhythm monitoring was not feasible, it remains unknown whether preoperative circadian AF patterns persisted after surgery in patients with an AF history. Future studies integrating continuous rhythm monitoring with autonomic and structural imaging are needed to validate AF chronotypes after cardiac surgery and to determine whether targeted or time-based interventions can modify AF trajectories.

## Conclusion

In the current study, AF after cardiac surgery emerged as a heterogeneous arrhythmia composed of distinct circadian subtypes that reflect differences in autonomic regulation and atrial remodeling. Rhythmic AF occurred in the majority of patients with sustained AF and was associated with a greater AF burden and more advanced structural remodeling, particularly in those with the mixed vagal–adrenergic type, compared with incidental or non-rhythmic AF. These findings suggest that sustained forms of AF after cardiac surgery frequently follow unique chronobiological trajectories rather than random onset dynamics. Future studies integrating rhythm monitoring with autonomic and structural assessments are warranted to validate these chronotypes and explore their therapeutic implications.

## Disclosures

US received consultancy fees or honoraria from Università della Svizzera Italiana (USI, Switzerland), Roche Diagnostics (Switzerland), EP Solutions Inc. (Switzerland), Johnson & Johnson Medical Limited (United Kingdom), Bayer Healthcare (Germany). US is the co-founder and shareholder of YourRhythmics BV, a spin-off company of the University of Maastricht. BM is a consultant for AtriCure and Medtronic. All other authors report no conflict of interests.
