# Supplementary material for: Circadian patterns of device-detected postoperative atrial fibrillation during 2.5 years of continuous monitoring after cardiac surgery: The RACE V–Tissue Bank Study
Source: Heart Rhythm O2. 2026 Jul 3;7(10):1884–99. doi: 10.1016/j.hroo.2026.06.023 (PMC13700383; doi:10.1016/j.hroo.2026.06.023)
Supplement: Supplementary file 1 [file mmc1.docx]

**Supplementary Material**

Overview

Supplementary Table	1	page 2

Supplementary Table 2	page 3

Supplementary Table 3	page 4

Supplementary Table 4	page 7

Supplementary Table 5	page 10

Supplementary Table 6	page 11

Supplementary Table 7	page 12

Supplementary Table 8	page 13

Supplementary Table 9	page 14

Supplementary Table 10	page 15

Supplementary Table 11	page 16

| **Supplementary Table 1. Incidence and occurrence patterns of atrial fibrillation according to AF subgroups** | | | | | |
| --- | --- | --- | --- | --- | --- |
| **Variable** | **Incidental AF***  **(n=27)** | **Non-Rhythmic AF (n=6)** | **Rhythmic AF (n=20)** | **P-value** | **Post-hoc group comparisons** |
| *AF incidence and patterns* | | | | | |
| AF burden, in % (IQR) | 0.05 (0.01-0.16) | 0.34 (0.14-4.40) | 1.23 (0.21-2.56) | **<0.001** | 0-1, 0-2 |
| AF density (IQR) | 0.98 (0.96-0.99) | 0.72 (0.48-0.91) | 0.84 (0.52-0.96) | **<0.001** | 0-1, 0-2 |
| AF-episodes per patient (IQR) | 4 (2-9) | 52 (39-61) | 61 (41-152) | **<0.001** | 0-1, 0-2 |
| AF-episodes ventricular rate (IQR) | 115 (100-138) | 96 (84-128) | 99 (92-110) | **0.027** | 0-2 |
| Median episode duration per patient (IQR) | 46 (4-220) | 12 (8-4736) | 8 (5-31) | 0.571 |  |
| Longest episode duration per patient (IQR) | 410 (34-1435) | 1501 (804-11179) | 1583 (619-10809) | **0.011** | 0-2 |
| *Early AF incidence and patterns* | | | | | |
| Early AF first 30 days (%) | 23 (85.2) | 6 (100) | 19 (95.0) | 0.368 |  |
| Early AF burden, in % (IQR) | 0.78 (0.01-3.63) | 6.48 (2.83-42.5) | 3.27 (0.45-6.84) | **0.023** | 0-1 |
| AF-episodes per patient (IQR) | 4 (2-6) | 31 (6-42) | 22 (9-41) | **<0.001** | 0-1, 0-2 |
| AF-episodes ventricular rate (IQR) | 122 (107-146) | 129 (100-133) | 97 (82-114) | **0.004** | 0-1, 0-2 |
| Median episode duration per patient (IQR) | 83 (12-367) | 22 (14-148) | 19 (5-76) | 0.054 |  |
| Longest episode duration per patient (IQR) | 259 (2-920) | 846 (369-12799) | 775 (104-905) | 0.158 |  |
| *Late AF incidence and patterns* | | | | | |
| Late AF after first 30 days (%) | 15 (55.6) | 6 (100) | 18 (90.0) | **0.009** | 0-1, 0-2 |
| Late AF burden, in % (IQR) | 0.003 (0.001-0.02) | 0.10 (0.004-3.11) | 0.16 (0.01-1.39) | **0.013** | 0-2 |
| AF-episodes per patient (IQR) | 2 (1-5) | 27 (8-42) | 23 (15-165) | **<0.001** | 0-2 |
| AF-episodes ventricular rate (IQR) | 111 (91-137) | 92 (82-125) | 99 (88-113) | 0.203 |  |
| Median episode duration per patient (IQR) | 6 (2-42) | 7 (3-170) | 8 (4-26) | 0.357 |  |
| Longest episode duration per patient (IQR) | 0 (0-26) | 710 (18-8727) | 349 (28-1067) | **<0.001** | 0-1, 0-2 |
| Bold values indicated significance at alpha threshold of 0.05. AF: atrial fibrillation, IQR: interquartile range  *Defined as less than 20 episodes in total | | | | | |

| **Supplementary Table 2. Incidence and occurrence patterns of atrial fibrillation according to chronotypes** | | | | | | |
| --- | --- | --- | --- | --- | --- | --- |
| **Variable** | **Non-Rhythmic AF (n=6)** | **Adrenergic AF (n=9)** | **Vagal AF (n=7)** | **Mixed Vagal-Adrenergic (n=4)** | **P-value** | **Post-hoc group comparisons** |
| *AF incidence and patterns* | | | | | | |
| AF burden, in % (IQR) | 0.34 (0.14-4.40) | 0.96 (0.16-2.55) | 0.77 (0.13-1.96) | 2.07 (1.24-70.3) | 0.400 |  |
| AF density (IQR) | 0.72 (0.48-0.91) | 0.85 (0.66-0.97) | 0.91 (0.48-0.97) | 0.64 (0.27-0.90) | 0.573 |  |
| AF-episodes per patient (IQR) | 52 (39-61) | 70 (36-81) | 42 (29-52) | 370 (233-680) | **0.023** | 0-3, 1-3 |
| AF-episodes ventricular rate (IQR) | 96 (84-128) | 109 (98-116) | 90 (70-118) | 97 (94-104) | 0.529 |  |
| Median episode duration per patient (IQR) | 12 (8-4736) | 8 (5-18) | 12 (8-37) | 34 (4-366) | 0.645 |  |
| Longest episode duration per patient (IQR) | 1501 (804-11179) | 2962 (866-5940) | 856 (354-8181) | 1747 (1309-11825) | 0.841 |  |
| *Early AF incidence and patterns* | | | | | | |
| Early AF first 30 days (%) | 6 (100) | 9 (100) | 6 (85.7) | 4 (100) | 0.420 |  |
| Early AF burden, in % (IQR) | 6.48 (2.83-42.5) | 2.82 (0.62-5.11) | 1.78 (0.001-7.35) | 10.71 (1.48-30.4) | 0.176 |  |
| AF-episodes per patient (IQR) | 31 (6-42) | 13 (3-41) | 19 (8-45) | 38 (20-71) | 0.732 |  |
| AF-episodes ventricular rate (IQR) | 129 (100-133) | 100 (82-107) | 87 (61-107) | 122 (98-123) | 0.089 |  |
| Median episode duration per patient (IQR) | 22 (14-148) | 24 (4-570) | 10 (6-398) | 34 (5-76) | 0.907 |  |
| Longest episode duration per patient (IQR) | 846 (369-12799) | 792 (249-926) | 648 (0-856) | 792 (136-1312) | 0.496 |  |
| *Late AF incidence and patterns* | | | | | | |
| Late AF after first 30 days (%) | 6 (100) | 8 (88.9) | 6 (85.7) | 4 (100) | 0.703 |  |
| Late AF burden, in % (IQR) | 0.10 (0.004-3.11) | 0.04 (0.006-0.31) | 0.31 (0.01-1.07) | 1.75 (1.14-71.3) | 0.104 |  |
| AF-episodes per patient (IQR) | 27 (8-42) | 19 (10-69) | 22 (12-192) | 344 (195-615) | **0.038** | 0-3, 1-3, 2-3 |
| AF-episodes ventricular rate (IQR) | 92 (82-125) | 109 (97-118) | 92 (83-118) | 97 (94-103) | 0.449 |  |
| Median episode duration per patient (IQR) | 7 (3-170) | 6 (4-11) | 20 (7-115) | 34 (4-479) | 0.534 |  |
| Longest episode duration per patient (IQR) | 710 (18-8727) | 68 (18-719) | 316 (20-1084) | 1328 (990-16755) | 0.160 |  |
| Bold values indicated significance at alpha threshold of 0.05. AF: atrial fibrillation, IQR: interquartile range  *Defined as less than 20 episodes in total | | | | | | |

| **Supplementary Table 3. Clinical characteristics of AF subgroups** | | | | | | |
| --- | --- | --- | --- | --- | --- | --- |
| **Variable** | **No AF**  **(n=45)** | **Incidental AF***  **(n=27)** | **Non-Rhythmic AF (n=6)** | **Rhythmic**  **AF (n=20)** | **P-value** | **Post-hoc group comparisons** |
| *General patient characteristics* | | | | | | |
| No AF-history (%) | 42 (93.3) | 19 (70.4) | 4 (66.7) | 6 (30.0) | **<0.001** | 0-3, 1-3, 0-2 |
| PAF-history (%) | 3 (6.7) | 3 (11.1) | 2 (33.3) | 8 (40.0) | **0.004** | 0-3, 1-3 |
| PerAF-history (%) | 0 (0) | 5 (18.5) | 0 (0) | 6 (30.0) | **0.002** | 0-3 |
| Any AF-history (%) | 3 (6.7) | 8 (29.6) | 2 (33.3) | 14 (70.0) | **<0.001** | 0-3, 1-3, 0-2 |
| Age, years (IQR) | 68 (59-72) | 68 (66-73) | 68 (63-73) | 68 (63-71) | 0.708 |  |
| Male sex (%) | 34 (75.6) | 21 (77.8) | 5 (83.3) | 15 (75.0) | 0.973 |  |
| BMI, kg/m^2^ (IQR) | 27.1 (24.9-29.1) | 26.2 (24.7-28.5) | 27.3 (26.3-29.4) | 28.9 (23.8-30.2) | 0.739 |  |
| NYHA class (IQR) | 2 (1-2) | 2 (2-2) | 2 (2-3) | 2 (2-3) | **0.031** | 0-3 |
| Hypertension (%) | 41 (91.1) | 26 (96.3) | 6 (100) | 17 (85.0) | 0.470 |  |
| Dyslipidemia (%) | 39 (86.7) | 24 (88.9) | 5 (83.3) | 17 (85.0) | 0.974 |  |
| Diabetes mellitus (%) | 9 (20.0) | 4 (14.8) | 0 (0) | 5 (25.0) | 0.523 |  |
| COPD (%) | 7 (15.6) | 4 (14.8) | 0 (0) | 2 (10.0) | 0.714 |  |
| Pulmonary hypertension (%) | 4 (8.9) | 5 (18.5) | 0 (0) | 4 (20.0) | 0.376 |  |
| Peripheral artery disease (%) | 10 (22.2) | 4 (14.8) | 3 (50.0) | 3 (15.0) | 0.240 |  |
| Creatinine, μmol/L (IQR) | 75.4 (60.0-85.2) | 70.5 (62.7-81.3) | 73.5 (63.8-90.0) | 72.9 (61.2-84.0) | 0.847 |  |
| Sleep apnea (%) | 2 (4.4) | 1 (3.7) | 0 (0) | 3 (15.0) | 0.304 |  |
| Tobacco use (%) | 31 (68.9) | 15 (55.6) | 3 (50.0) | 11 (55.0) | 0.547 |  |
| Alcohol use (%) | 29 (64.4) | 15 (55.6) | 4 (66.7) | 11 (55.0) | 0.823 |  |
| CHA₂DS₂-VASc-score (IQR) | 3 (2-4) | 3 (3-4) | 3 (3-5) | 3 (2-4) | 0.496 |  |
| *Preoperative transthoracic echocardiography* | | | | | | |
| LVEF, % (IQR) | 56 (50-60) | 56 (52-60) | 59 (55-65) | 59 (51-64) | 0.233 |  |
| LAVImax, ml/m^2^ (IQR) | 30.0 (25.8-38.3) | 38.9 (28.6-53.8) | 35.8 (28.1-44.4) | 40.4 (36.6-68.8) | **0.001** | 0-1, 0-3 |
| LAVImin, ml/m^2^ (IQR) | 12.0 (8.9-18.8) | 19.2 (11.3-29.1) | 15.0 (12.1-29.3) | 29.9 (17.3-49.4) | **<0.001** | 0-3 |
| LATEF, % (IQR) | 58.1 (51.3-66.9) | 56.2 (45.8-61.2) | 50.9 (37.6-58.7) | 36.8 (22.6-51.9) | **0.001** | 0-3, 1-3 |
| RAVImax, ml/m^2^ (IQR) | 21.2 (17.1-30.1) | 30.0 (25.0-36.4) | 33.1 (25.4-41.3) | 34.3 (26.4-41.6) | **<0.001** | 0-1, 0-3 |
| RAVImin, ml/m^2^ (IQR) | 9.9 (7.6-14.8) | 14.6 (11.9-18.7) | 14.7 (10.5-20.5) | 20.2 (13.8-24.0) | **<0.001** | 0-1, 0-3 |
| RATEF, % (IQR) | 54.1 (42.9-60.1) | 46.5 (35.5-56.6) | 53.3 (44.3-66.4) | 48.2 (25.0-51.2) | 0.116 |  |
| LVM, grams (IQR) | 193 (146-251) | 204 (158-283) | 194 (165-252) | 203 (165-240) | 0.864 |  |
| LVMI, grams/m^2^ (IQR) | 93 (77-128) | 97 (81-134) | 98 (76-130) | 94 (85-122) | 0.938 |  |
| E-wave, cm/s (IQR) | 69 (59-83) | 76 (61-106) | 64 (52-79) | 84 (64-121) | 0.130 |  |
| *Preoperative 12-leads electrocardiography* | | | | | | |
| PR-interval, msec (IQR) | 158 (146-178) | 172 (146-185) | 158 (148-194) | 184 (166-210) | **0.033** | 0-3 |
| P-wave duration, msec (IQR) | 108 (104-120) | 116 (110-128) | 118 (116-124) | 120 (108-136) | 0.088 |  |
| QRS-duration, msec (IQR) | 92 (82-104) | 98 (90-104) | 102 (82-118) | 97 (89-104) | 0.647 |  |
| QT-interval, msec (IQR) | 406 (379-427) | 402 (388-426) | 401 (371-439) | 388 (372-430) | 0.796 |  |
| QTc-interval, msec (IQR) | 422 (411-446) | 416 (402-447) | 417 (388-442) | 432 (414-456) | 0.399 |  |
| *Preoperative biomarkers* | | | | | | |
| ANG2, ng/ml (IQR) | 1.87 (1.58-2.38) | 2.14 (1.76-2.50) | 1.88 (1.46-2.64) | 3.15 (1.52-5.40) | 0.300 |  |
| BMP10, ng/ml (IQR) | 1.71 (1.59-1.94) | 1.99 (1.74-2.31) | 1.80 (1.50-2.13) | 1.93 (1.73-2.52) | **0.014** | 0-1, 0-3 |
| DKK3, ng/ml (IQR) | 52.4 (46.3-59.8) | 55.7 (52.7-65.1) | 50.4 (42.1-57.9) | 55.6 (48.5-62.1) | 0.221 |  |
| ESM1, pg/ml (IQR) | 1775 (1520-2178) | 1930 (1707-2594) | 1639 (1525-1890) | 2409 (1545-2785) | 0.171 |  |
| FGF23, ng/ml (IQR) | 145.8 (109.8-196.2) | 142.2 (125.2-189.6) | 172.0 (159.4-219.8) | 154.2 (128.3-208.5) | 0.362 |  |
| IGFBP7, ng/ml (IQR) | 99.6 (88.9-108.4) | 106.5 (94.7-119.6) | 101.1 (99.0-121.7) | 104.5 (91.0-117.0) | 0.349 |  |
| NT-proBNP, pg/ml (IQR) | 754 (447-1595) | 1771 (706-2907) | 1626 (1042-4280) | 1936 (601-3363) | 0.055 |  |
| GDF15, pg/ml (IQR) | 1533 (856-2143) | 1280 (1085-2379) | 1512 (957-2763) | 1356 (997-1873) | 0.982 |  |
| IL6, pg/ml (IQR) | 3.5 (1.9-7.7) | 4.2 (2.6-8.7) | 3.2 (2.2-5.3) | 2.5 (1.8-6.4) | 0.591 |  |
| Pro-BNP2, pg/ml (IQR) | 145 (82-581) | 428 (148-774) | 296 (150-1785) | 533 (127-1096) | 0.121 |  |
| Troponin T, pg/ml (IQR) | 14.2 (10.2-22.4) | 13.8 (9.9-19.1) | 13.6 (10.6-41.7) | 13.3 (10.4-19.0) | 0.990 |  |
| *Surgical data* | | | | | | |
| Type of operation (%) | 45 (100) | 27 (100) | 6 (100) | 20 (100) | **0.033** | 0-1, 0-3 |
| CABG (%) | 37 (83.3) | 16 (59.3) | 5 (83.3) | 9 (45.0) | **0.013** | 0-3 |
| AVR (%) | 5 (11.1) | 3 (11.1) | 1 (16.7) | 4 (20.0) | 0.630 |  |
| MV surgery (%) | 3 (6.7) | 8 (29.6) | 0 (0) | 7 (35.0) | **0.010** | 0-3 |
| Rhythm surgery (%) | 2 (4.4) | 7 (25.9) | 1 (16.6) | 13 (65.0) | **<0.001** | 0-1, 0-3, 1-3, 2-3 |
| PVI (%) | 0 (0) | 3 (11.1) | 1 (16.6) | 3 (15.0) | 0.079 |  |
| Left Maze (%) | 2 (4.4) | 4 (14.8) | 0 (0) | 6 (30.0) | **0.024** | 0-3, 2-3 |
| Full Cox Maze (%) | 0 (0) | 0 (0) | 0 (0) | 4 (20.0) | **0.001** | 0-3, 1-3, 2-3 |
| *Postoperative data* | | | | | | |
| Rhythm control during admission (%) | 2 (4.4) | 7 (25.9) | 1 (16.7) | 5 (25.0) | **0.048** | 0-1, 0-3 |
| AAD at discharge (%) | 2 (4.4) | 3 (11.1) | 0 (0) | 4 (20.0) | 0.192 |  |
| Sotalol at discharge (%) | 2 (4.4) | 3 (11.1) | 0 (0) | 3 (15.0) | 0.404 |  |
| Amiodarone at discharge (%) | 0 (0) | 0 (0) | 0 (0) | 1 (5.0) | 0.268 |  |
| (D)OAC at discharge (%) | 5 (11.4) | 16 (61.5) | 5 (83.3) | 17 (89.5) | **<0.001** | 0-1, 0-2, 0-3 |
| Diuretics at discharge (%) | 13 (28.9) | 13 (48.1) | 3 (50.0) | 14 (70.0) | **0.019** | 0-3, 1-3 |
| Beta blockers at discharge (%) | 40 (88.9) | 26 (96.3) | 6 (100) | 16 (80.0) | 0.255 |  |
| Bold values indicated significance at alpha threshold of 0.05.  ANG-2: angiopoietin 2, AVR: aortic valve replacement, BMI: body mass index, BMP-10: bone morphogenic protein 10, CABG: coronary artery bypass grafting, COPD: chronic obstructive pulmonary disease, DKK-3: dickkopf 3, ESM-1: endothelial cell-specific molecule 1, FGF-23: fibroblast growth factor 23, GDF-15: growth differentiation factor 15, ICU: intensive care unit, IGFBP-7: insulin-like growth factor-binding protein 7, IL-6: interleukin 6, IQR: interquartile range, LATEF: left atrial total ejection fraction, LAVI: left atrial volume indexed for body surface area, LVEF: left ventricle ejection fraction, LVMI: left ventricular mass indexed for body surface area, MV: mitral valve, ng: nanogram, NT-proBNP: n-terminal brain natriuretic peptide, NYHA: New York Heart Association, pg: picogram, pro-BNP2: pro brain natriuretic peptide 2, PVI: pulmonary vein isolation, RATEF: right atrial total ejection fraction, RAVI: right atrial volume indexed for body surface area, SD: standard deviation, SR: sinus rhythm.  *Defined as less than 20 episodes in total | | | | | | |

| **Supplementary Table 4. Clinical characteristics of AF chronotypes** | | | | | | |
| --- | --- | --- | --- | --- | --- | --- |
| **Variable** | **Non-Rhythmic AF (n=6)** | **Adrenergic**  **AF (n=9)** | **Vagal**  **AF (n=7)** | **Mixed Adrenergic-Vagal (n=4)** | **P-value** | **Post-hoc group comparisons** |
| *General patient characteristics* | | | | | | |
| No AF-history (%) | 4 (66.7) | 3 (33.3) | 2 (28.6) | 1 (25.0) | 0.438 |  |
| PAF-history (%) | 2 (33.3) | 3 (33.3) | 3 (42.9) | 2 (50.0) | 0.930 |  |
| PerAF-history (%) | 0 (0) | 3 (33.3) | 2 (28.6) | 1 (25.0) | 0.482 |  |
| Any AF-history (%) | 2 (33.3) | 6 (66.7) | 5 (71.4) | 3 (75.0) | 0.438 |  |
| Age, years (IQR) | 68 (63-73) | 68 (63-72) | 67 (61-71) | 69 (62-71) | 0.900 |  |
| Male sex (%) | 5 (83.3) | 7 (77.8) | 6 (85.7) | 2 (50.0) | 0.556 |  |
| BMI, kg/m^2^ (IQR) | 27.3 (26.3-29.4) | 24.6 (22.5-30.2) | 29.4 (25.3-30.3) | 29.4 (24.5-31.8) | 0.674 |  |
| NYHA class (IQR) | 2 (2-3) | 2 (1-2) | 2 (2-3) | 3 (2-3) | 0.274 |  |
| Hypertension (%) | 6 (100) | 7 (77.8) | 6 (85.7) | 4 (100) | 0.501 |  |
| Dyslipidemia (%) | 5 (83.3) | 6 (66.7) | 7 (100) | 4 (100) | 0.237 |  |
| Diabetes mellitus (%) | 0 (0) | 2 (22.2) | 2 (28.6) | 1 (25.0) | 0.581 |  |
| COPD (%) | 0 (0) | 1 (11.1) | 0 (0) | 1 (25.0) | 0.404 |  |
| Pulmonary hypertension (%) | 0 (0) | 1 (11.1) | 2 (28.6) | 1 (25.0) | 0.487 |  |
| Peripheral artery disease (%) | 3 (50.0) | 1 (11.1) | 1 (14.3) | 1 (25.0) | 0.322 |  |
| Creatinine, μmol/L (IQR) | 73.5 (63.8-90.0) | 71.2 (55.8-81.6) | 72.9 (60.0-90.0) | 75.5 (66.7-81.9) | 0.850 |  |
| Sleep apnea (%) | 0 (0) | 3 (33.3) | 0 (0) | 0 (0) | 0.093 |  |
| Tobacco use (%) | 3 (50.0) | 5 (55.6) | 3 (42.9) | 3 (75.0) | 0.775 |  |
| Alcohol use (%) | 4 (66.7) | 6 (66.7) | 4 (57.1) | 1 (25.0) | 0.523 |  |
| CHA₂DS₂-VASc-score (IQR) | 3 (3-5) | 3 (2-5) | 3 (3-4) | 3 (2-4) | 0.789 |  |
| *Preoperative transthoracic echocardiography* | | | | | | |
| LVEF, % (IQR) | 59 (55-65) | 55 (53-60) | 60 (50-64) | 60 (55-65) | 0.503 |  |
| LAVImax, ml/m^2^ (IQR) | 35.8 (28.1-44.4) | 42.9 (38.4-62.8) | 49.3 (28.5-92.2) | 35.6 (34.5-64.9) | 0.220 |  |
| LAVImin, ml/m^2^ (IQR) | 15.0 (12.1-29.3) | 30.4 (19.6-38.4) | 47.3 (18.7-64.8) | 23.1 (14.4-44.3) | 0.150 |  |
| LATEF, % (IQR) | 50.9 (37.6-58.7) | 36.9 (22.5-57.7) | 33.9 (22.2-47.7) | 41.2 (23.3-59.1) | 0.493 |  |
| RAVImax, ml/m^2^ (IQR) | 33.1 (25.4-41.3) | 28.1 (24.5-36.0) | 53.6 (30.9-70.8) | 35.3 (28.6-37.6) | **0.049** | 1-2 |
| RAVImin, ml/m^2^ (IQR) | 14.7 (10.5-20.5) | 17.2 (13.8-22.4) | 26.6 (15.3-62.4) | 18.4 (11.4-22.6) | 0.070 |  |
| RATEF, % (IQR) | 53.3 (44.3-66.4) | 49.3 (23.6-50.9) | 47.2 (11.6-51.3) | 50.7 (35.5-60.9) | 0.206 |  |
| LVM, grams (IQR) | 194 (165-252) | 238 (177-271) | 201 (162-207) | 159 (124-213 | 0.307 |  |
| LVMI, grams/m^2^ (IQR) | 98 (76-130) | 114 (87-127) | 92 (84-103) | 86 (64-94) | 0.296 |  |
| E-wave, cm/s (IQR) | 64 (52-79) | 83 (71-102) | 126 (64-160) | 75 (53-129) | 0.271 |  |
| *Preoperative 12-leads electrocardiography* | | | | | | |
| PR-interval, msec (IQR) | 158 (148-194) | 175 (157-192) | 210 (188-225) | 186 (174-202) | 0.169 |  |
| P-wave duration, msec (IQR) | 118 (116-124) | 120 (117-134) | 116 (112-128) | 118 (100-136) | 0.876 |  |
| QRS-duration, msec (IQR) | 102 (82-118) | 98 (84-108) | 96 (90-134) | 95 (91-100) | 0.929 |  |
| QT-interval, msec (IQR) | 401 (371-439) | 390 (372-430) | 388 (370-430) | 406 (318-450) | 0.985 |  |
| QTc-interval, msec (IQR) | 417 (388-442) | 430 (418-462) | 434 (398-462) | 430 (408-439) | 0.490 |  |
| *Preoperative biomarkers* | | | | | | |
| ANG2, ng/ml (IQR) | 1.88 (1.46-2.64) | 2.83 (1.58-3.91) | 5.40 (1.62-7.51) | 2.59 (1.52-3.53) | 0.485 |  |
| BMP10, ng/ml (IQR) | 1.80 (1.50-2.13) | 1.92 (1.73-2.26) | 2.27 (1.70-3.08) | 1.96 (1.75-2.50) | 0.686 |  |
| DKK3, ng/ml (IQR) | 50.4 (42.1-57.9) | 51.8 (43.8-58.6) | 59.6 (48.9-63.8) | 64.7 (47.3-81.9) | 0.562 |  |
| ESM1, pg/ml (IQR) | 1639 (1525-1890) | 2034 (1659-2531) | 2502 (1676-2876) | 3567 (1732-4775) | 0.319 |  |
| FGF23, ng/ml (IQR) | 172.0 (159.4-219.8) | 154.7 (119.1-180.2) | 150.4 (124.1-197.8) | 181.4 (147.6-369.0) | 0.588 |  |
| IGFBP7, ng/ml (IQR) | 101.1 (99.0-121.7) | 96.6 (89.4-112.5) | 106.7 (100.4-117.4) | 107.9 (95.0-120.5) | 0.704 |  |
| NT-proBNP, pg/ml (IQR) | 1626 (1042-4280) | 1462 (565-2617) | 2838 (1960-3119) | 2404 (453-4285) | 0.628 |  |
| GDF15, pg/ml (IQR) | 1512 (957-2763) | 1433 (926-2306) | 1296 (927-1691) | 1350 (1251-2713) | 0.892 |  |
| IL6, pg/ml (IQR) | 3.2 (2.2-5.3) | 5.2 (2.0-6.4) | 2.1 (1.8-2.7) | 2.5 (2.1-4.5) | 0.684 |  |
| Pro-BNP2, pg/ml (IQR) | 296 (150-1785) | 495 (124-794) | 1008 (270-1097) | 629 (66-1368) | 0.785 |  |
| Troponin T, pg/ml (IQR) | 13.6 (10.6-41.7) | 16.4 (8.9-27.4) | 13.3 (9.7-17.1) | 12.5 (10.8-17.5) | 0.940 |  |
| *Surgical data* | | | | | | |
| Type of operation (%) | 6 (100) | 9 (100) | 7 (100) | 4 (100) | 0.227 |  |
| CABG (%) | 5 (83.3) | 4 (44.4) | 2 (28.6) | 3 (75.0) | 0.176 |  |
| AVR (%) | 1 (16.7) | 3 (33.3) | 1 (14.3) | 0 (0) | 0.524 |  |
| MV surgery (%) | 0 (0) | 2 (22.2) | 4 (57.1) | 1 (25.0) | 0.135 |  |
| Rhythm surgery (%) | 1 (16.6) | 6 (66.6) | 4 (57.2) | 3 (75.0) | 0.197 |  |
| PVI (%) | 1 (16.6) | 1 (11.1) | 0 (0) | 2 (50.0) | *NA* |  |
| Left Maze (%) | 0 (0) | 4 (44.4) | 2 (28.6) | 0 (0) | *NA* |  |
| Full Cox Maze (%) | 0 (0) | 1 (11.1) | 2 (28.6) | 1 (25.0) | *NA* |  |
| *Postoperative data* | | | | | | |
| Rhythm control during admission (%) | 1 (16.7) | 2 (22.2) | 0 (0) | 3 (75.0) | **0.040** | 2-3 |
| AAD at discharge (%) | 0 (0) | 2 (22.2) | 0 (0) | 2 (50.0) | 0.095 |  |
| Sotalol at discharge (%) | 0 (0) | 1 (11.1) | 0 (0) | 2 (50.0) | 0.189 |  |
| Amiodarone at discharge (%) | 0 (0) | 1 (11.1) | 0 (0) | 0 (0) | 0.580 |  |
| (D)OAC at discharge (%) | 5 (83.3) | 8 (88.9) | 5 (83.3) | 4 (100) | 0.850 |  |
| Diuretics at discharge (%) | 3 (50.0) | 6 (66.7) | 5 (71.4) | 3 (75.0) | 0.823 |  |
| Beta blockers at discharge (%) | 6 (100) | 8 (88.9) | 5 (71.4) | 3 (75.0) | 0.487 |  |
| Bold values indicated significance at alpha threshold of 0.05.  AAD: antiarrhythmic drug, ANG-2: angiopoietin 2, AVR: aortic valve replacement, BMI: body mass index, BMP-10: bone morphogenic protein 10, CABG: coronary artery bypass grafting, COPD: chronic obstructive pulmonary disease, DKK-3: dickkopf 3, ESM-1: endothelial cell-specific molecule 1, FGF-23: fibroblast growth factor 23, GDF-15: growth differentiation factor 15, ICU: intensive care unit, IGFBP-7: insulin-like growth factor-binding protein 7, IL-6: interleukin 6, IQR: interquartile range, LATEF: left atrial total ejection fraction, LAVI: left atrial volume indexed for body surface area, LVEF: left ventricle ejection fraction, LVMI: left ventricular mass indexed for body surface area, MV: mitral valve, ng: nanogram, NT-proBNP: n-terminal brain natriuretic peptide, NYHA: New York Heart Association, pg: picogram, pro-BNP2: pro brain natriuretic peptide 2, PVI: pulmonary vein isolation, RATEF: right atrial total ejection fraction, RAVI: right atrial volume indexed for body surface area, SD: standard deviation, SR: sinus rhythm.  *Defined as less than 20 episodes in total | | | | | | |

| **Supplementary Table 5. Histological characteristics of AF subgroups** | | | | | | |
| --- | --- | --- | --- | --- | --- | --- |
| **Variable** | **No AF (n=45)** | **Incidental AF***  **(n=27)** | **Non-Rhythmic AF (n=6)** | **Rhythmic AF (n=20)** | **P-value** | **Post-hoc group comparisons** |
| *Left atrial characteristics* | | | | | | |
| Myocyte diameter, in μm (IQR) | 10.7 (10.1-11.6) | 11.1 (10.9-11.8) | 11.2 (10.1-11.5) | 12.2 (11.2-13.5) | **0.005** | 0-3, 1-3 |
| EMF, in μm (IQR) | 4.9 (4.3-5.6) | 5.1 (4.6-5.5) | 5.0 (4.5-5.5) | 5.3 (4.8-5.9) | 0.721 |  |
| ECM, in % (IQR) | 23.2 (21.1-25.3) | 20.7 (18.7-23.9) | 24.2 (23.2-26.3) | 19.9 (18.1-26.2) | 0.057 |  |
| Capillary density, ratio (IQR) | 0.23 (0.19-0.31) | 0.20 (0.18-0.26) | 0.21 (0.13-0.27) | 0.31 (0.24-0.55) | **0.011** | 1-3 |
| Capillary size, in μm (IQR) | 4.21 (3.81-4.70) | 4.47 (3.71-4.76) | 4.54 (4.27-4.89) | 4.15 (3.59-4.92) | 0.762 |  |
| Fibroblast density, ratio (IQR) | 0.47 (0.38-0.55) | 0.48 (0.41-0.61) | 0.43 (0.41-0.44) | 0.60 (0.44-0.68) | 0.134 |  |
| *Right atrial characteristics* | | | | | | |
| Myocyte diameter, in μm (IQR) | 10.8 (9.9-11.4) | 11.5 (10.8-11.9) | 10.9 (9.7-11.1) | 11.4 (10.8-12.4) | 0.075 |  |
| EMF, in μm (IQR) | 6.0 (5.4-6.6) | 6.3 (5.6-6.7) | 5.9 (5.8-6.0) | 6.8 (6.4-7.2) | **0.022** | 0-3, 1-3, 2-3 |
| ECM, in % (IQR) | 25.9 (22.9-27.9) | 25.4 (21.5-28.4) | 25.5 (24.0-26.6) | 25.5 (21.9-27.9) | 0.954 |  |
| Capillary density, ratio (IQR) | 0.30 (0.25-0.35) | 0.31 (0.26-0.32) | 0.25 (0.22-0.37) | 0.29 (0.20-0.48) | 0.998 |  |
| Capillary size, in μm (IQR) | 3.72 (3.22-4.05) | 4.11 (3.61-4.38) | 4.02 (3.89-4.92) | 3.84 (3.26-4.54) | 0.332 |  |
| Fibroblast density, ratio (IQR) | 0.51 (0.40-0.58) | 0.53 (0.43-0.65) | 0.48 (0.45-0.66) | 0.51 (0.44-0.80) | 0.792 |  |
| Bold values indicated significance at alpha threshold of 0.05.  AF: atrial fibrillation, SD: standard deviation, μm: micrometer  *Defined as less than 20 episodes in total | | | | | | |

| **Supplementary Table 6. Histological characteristics of AF chronotypes** | | | | | | |
| --- | --- | --- | --- | --- | --- | --- |
| **Variable** | **Non-Rhythmic AF (n=6)** | **Adrenergic AF (n=9)** | **Vagal AF (n=7)** | **Mixed Adrenergic-Vagal (n=4)** | **P-value** | **Post-hoc group comparisons** |
| *Left atrial characteristics* | | | | | | |
| Myocyte diameter, in μm (IQR) | 11.2 (10.1-11.5) | 12.7 (11.3-13.8) | 12.0 (11.2-12.6) | 12.6 (10.2-17.4) | 0.087 |  |
| EMF, in μm (IQR) | 5.0 (4.5-5.5) | 4.9 (4.6-6.3) | 6.2 (5.2-7.0) | 5.8 (4.7-6.9) | 0.724 |  |
| ECM, in % (IQR) | 24.2 (23.2-26.3) | 18.5 (16.9-20.7) | 22.4 (18.9-25.5) | 24.7 (18.4-28.3) | 0.140 |  |
| Capillary density, ratio (IQR) | 0.21 (0.13-0.27) | 0.42 (0.31-0.57) | 0.30 (0.22-0.30) | 0.38 (0.23-0.51) | 0.103 |  |
| Capillary size, in μm (IQR) | 4.54 (4.27-4.89) | 4.06 (3.22-4.15) | 4.92 (3.72-5.05) | 4.23 (3.89-5.02) | 0.268 |  |
| Fibroblast density, ratio (IQR) | 0.43 (0.41-0.44) | 0.62 (0.45-0.63) | 0.58 (0.47-0.64) | 0.70 (0.42-0.90) | 0.421 |  |
| *Right atrial characteristics* | | | | | | |
| Myocyte diameter, in μm (IQR) | 10.9 (9.7-11.1) | 11.2 (10.8-11.6) | 11.7 (9.1-13.0) | 11.5 (10.8-12.0) | 0.271 |  |
| EMF, in μm (IQR) | 5.9 (5.8-6.0) | 6.4 (6.0-6.8) | 6.8 (6.3-7.3) | 9.6 (7.2-20.7) | **0.009** | 0-3, 1-3 |
| ECM, in % (IQR) | 25.5 (24.0-26.6) | 23.8 (21.4-25.4) | 24.9 (23.0-27.6) | 27.8 (24.8-30.2) | 0.258 |  |
| Capillary density, ratio (IQR) | 0.25 (0.22-0.37) | 0.36 (0.29-0.59) | 0.25 (0.15-0.45) | 0.27 (0.24-0.28) | 0.798 |  |
| Capillary size, in μm (IQR) | 4.02 (3.89-4.92) | 3.76 (3.00-4.49) | 4.52 (3.39-4.58) | 3.59 (3.45-3.84) | 0.694 |  |
| Fibroblast density, ratio (IQR) | 0.48 (0.45-0.66) | 0.65 (0.45-1.05) | 0.52 (0.50-0.75) | 0.50 (0.46-0.53) | 0.827 |  |
| Bold values indicated significance at alpha threshold of 0.05.  AF: atrial fibrillation, SD: standard deviation, μm: micrometer | | | | | | |

| **Supplementary Table 7. Paired difference in histological characteristics of AF chronotypes** | | | | | | |
| --- | --- | --- | --- | --- | --- | --- |
| **Variable** | **Non-Rhythmic AF (n=6)** | **Adrenergic AF (n=9)** | **Vagal AF**  **(n=7)** | **Mixed Vagal-Adrenergic (n=4)** | **P-value** | **Post-hoc group comparisons** |
| *Δ Atrial characteristics (RA appendage – LA appendage)* | | | | | | |
| Myocyte diameter, in μm (IQR) | -0.30 (-0.94 to -0.13) | -1.33 (-1.89 to -0.58) | 0.15 (-1.01 to 0.60) | 0.76 (-6.42 to 10.48) | 0.657 |  |
| EMF, in μm (IQR) | 0.66 (0.35 to 0.87) | 0.87 (0.22 to 1.66) | 0.88 (0.79 to 1.71) | 4.74 (2.11 to 15.0) | **0.042** | 0-3, 1-3, 2-3 |
| ECM, in % (IQR) | 0.82 (-0.79 to 3.74) | 4.35 (3.92 to 5.37) | -0.11 (-3.12 to 3.14) | 1.44 (0.09 to 8.17) | 0.186 |  |
| Capillary density, ratio (IQR) | 0.08 (0.02 to 0.28) | 0.05 (-0.24 to 0.34) | -0.07 (-0.13 to 0.13) | -0.17 (-0.25 to -0.03) | 0.505 |  |
| Capillary size, in μm (IQR) | -0.48 (-0.82 to -0.15) | -0.06 (-0.46 to 0.22) | -0.05 (-0.31 to 0.42) | -0.20 (-0.54 to -0.10) | 0.358 |  |
| Fibroblast density, ratio (IQR) | 0.14 (0.05 to 0.36) | 0.25 (-0.32 to 0.82) | 0.07 (-0.09 to 0.48) | -0.32 (-0.33 to -0.08) | 0.480 |  |
| Bold values indicated significance at alpha threshold of 0.05.  AF: atrial fibrillation, SD: standard deviation, μm: micrometer | | | | | | |

| **Supplementary Table 8. Progression patterns of atrial fibrillation according to AF subgroups** | | | | |
| --- | --- | --- | --- | --- |
| **Variable** | **Non-Rhythmic AF (n=6)** | **Rhythmic AF (n=20)** | **P-value** | **Post-hoc group comparisons** |
| *AF burden* | | | | |
| Overall (%) | 6 (100) | 20 (100) | 0.148 |  |
| Stable (%) | 4 (66.7) | 5 (25.0) | *NA* |  |
| Decreasing (%) | 2 (33.3) | 12 (60.0) | *NA* |  |
| Increasing (%) | 0 (0) | 3 (15.0) | *NA* |  |
| *Circadian phase* | | | | |
| Overall (%) | *NA* | 20 (100) | *NA* |  |
| Stable (%) | *NA* | 14 (70.0) | *NA* |  |
| Drifting rhythm (%) | *NA* | 6 (30.0) | *NA* |  |
| *Median ventricular rate* | | | | |
| Overall (%) | 6 (100) | 20 (100) | 0.492 |  |
| Stable (%) | 6 (100) | 16 (80.0) | *NA* |  |
| Decreasing (%) | 0 (0) | 3 (15.0) | *NA* |  |
| Increasing (%) | 0 (0) | 1 (5.0) | *NA* |  |
| Bold values indicated significance at alpha threshold of 0.05.  AF: atrial fibrillation, IQR: interquartile range | | | | |

| **Supplementary Table 9. Progression patterns of atrial fibrillation according to AF chronotypes** | | | | | | |
| --- | --- | --- | --- | --- | --- | --- |
| **Variable** | **Non-Rhythmic AF (n=6)** | **Adrenergic AF (n=9)** | **Vagal AF (n=7)** | **Mixed Adrenergic-Vagal (n=4)** | **P-value** | **Post-hoc group comparisons** |
| *AF burden* | | | | | | |
| Overall (%) | 6 (100) | 9 (100) | 7 (100) | 4 (100) | 0.610 |  |
| Stable (%) | 4 (66.7) | 2 (22.2) | 2 (28.6) | 1 (25.0) | 0.307 |  |
| Decreasing (%) | 2 (33.3) | 6 (66.7) | 4 (57.1) | 2 (50.0) | 0.282 |  |
| Increasing (%) | 0 (0) | 1 (11.1) | 1 (14.3) | 1 (25.0) | 0.672 |  |
| *Circadian phase* | | | | | | |
| Overall (%) | *NA* | 9 (100) | 7 (100) | 4 (100) | **0.032** | 2-3 |
| Stable (%) | *NA* | 6 (66.7) | 7 (100) | 1 (25.0) | *NA* |  |
| Drifting rhythm (%) | *NA* | 3 (33.3) | 0 (0) | 3 (75.0) | *NA* |  |
| *Median ventricular rate* | | | | | | |
| Overall (%) | 6 (100) | 9 (100) | 7 (100) | 4 (100) | 0.596 |  |
| Stable (%) | 6 (100) | 6 (66.7) | 6 (85.7) | 4 (100) | 0.256 |  |
| Decreasing (%) | 0 (0) | 2 (22.2) | 1 (14.3) | 0 (0) | 0.501 |  |
| Increasing (%) | 0 (0) | 1 (11.1) | 0 (0) | 0 (0) | 0.580 |  |
| Bold values indicated significance at alpha threshold of 0.05.  AF: atrial fibrillation, IQR: interquartile range | | | | | | |

| **Supplementary Table 10. Adverse events for AF subgroups** | | | | | | |
| --- | --- | --- | --- | --- | --- | --- |
| **Variable** | **No AF (n=45)** | **Incidental AF***  **(n=27)** | **Non-Rhythmic AF (n=6)** | **Rhythmic AF (n=20)** | **P-value** | **Post-hoc group comparisons** |
| Cumulative any events (IQR) | 0 (0-1 | 0 (0-1) | 0 (0-1) | 0 (0-2) | 0.338 |  |
| Cumulative cardiovascular events (IQR) | 0 (0-1) | 0 (0-0) | 0 (0-1) | 0 (0-1) | 0.195 |  |
| Ischemic stroke (%) | 1 (2.2) | 0 (0) | 0 (0) | 1 (5.0) | 0.666 |  |
| Hemorrhagic stroke (%) | 0 (0) | 0 (0) | 0 (0) | 0 (0) | *NA* |  |
| Systemic embolism (%) | 0 (0) | 1 (3.7) | 0 (0) | 0 (0) | 0.448 |  |
| Vascular interventions (%) | 1 (2.2) | 3 (11.1) | 1 (16.7) | 0 (0) | 0.138 |  |
| Major bleeding (%) | 0 (0) | 0 (0) | 0 (0) | 0 (0) | *NA* |  |
| Myocardial infarction (%) | 1 (2.2) | 0 (0) | 0 (0) | 0 (0) | 0.755 |  |
| Heart failure hospitalization (%) | 1 (2.2) | 0 (0) | 0 (0) | 0 (0) | 0.755 |  |
| PM implantation (%) | 1 (2.2) | 0 (0) | 0 (0) | 0 (0) | 0.755 |  |
| ICD implantation (%) | 1 (2.2) | 1 (3.7) | 0 (0) | 0 (0) | 0.820 |  |
| ER visits for arrhythmia (%) | 2 (4.4) | 0 (0) | 0 (0) | 6 (30.0) | **<0.001** | 0-3, 1-3 |
| Electrical cardioversion (%) | 0 (0) | 0 (0) | 1 (16.7) | 4 (20.0) | **0.002** | 0-3, 1-3, 1-2, 0-2 |
| Pulmonary vein ablation (%) | 0 (0) | 0 (0) | 1 (16.7) | 2 (10.0) | **0.026** | 0-3, 0-2, 1-2 |
| ER visits for any cause (%) | 11 (24.4) | 4 (14.8) | 3 (50.0) | 10 (50.0) | **0.033** | 0-3, 1-3 |
| Bold values indicated significance at alpha threshold of 0.05.  ER: emergency room, ICD: implantable cardioverter defibrillator, PM: pacemaker.  *Defined as less than 20 episodes in total | | | | | | |

| **Supplementary Table 11. Adverse events for AF chronotypes** | | | | | | |
| --- | --- | --- | --- | --- | --- | --- |
| **Variable** | **Non-Rhythmic AF (n=6)** | **Adrenergic AF (n=9)** | **Vagal AF (n=7)** | **Mixed Adrenergic-Vagal (n=4)** | **P-value** | **Post-hoc group comparisons** |
| Cumulative any events (IQR) | 0 (0-1) | 1 (0-2) | 0 (0-1) | 1 (0-2) | 0.786 |  |
| Cumulative cardiovascular events (IQR) | 0 (0-1) | 0 (0-2) | 0 (0-1) | 0 (0-1) | 0.564 |  |
| Ischemic stroke (%) | 0 (0) | 1 (11.1) | 0 (0) | 0 (0) | 0.580 |  |
| Hemorrhagic stroke (%) | 0 (0) | 0 (0) | 0 (0) | 0 (0) | *NA* |  |
| Systemic embolism (%) | 0 (0) | 0 (0) | 0 (0) | 0 (0) | *NA* |  |
| Vascular interventions (%) | 1 (16.7) | 0 (0) | 0 (0) | 0 (0) | 0.325 |  |
| Major bleeding (%) | 0 (0) | 0 (0) | 0 (0) | 0 (0) | *NA* |  |
| Myocardial infarction (%) | 0 (0) | 0 (0) | 0 (0) | 0 (0) | *NA* |  |
| Heart failure hospitalization (%) | 0 (0) | 0 (0) | 0 (0) | 0 (0) | *NA* |  |
| PM implantation (%) | 0 (0) | 0 (0) | 0 (0) | 0 (0) | *NA* |  |
| ICD implantation (%) | 0 (0) | 0 (0) | 0 (0) | 0 (0) | *NA* |  |
| ER visits for arrhythmia (%) | 0 (0) | 5 (55.6) | 1 (14.3) | 0 (0) | **0.034** | 0-1, 1-2, 1-3 |
| Electrical cardioversion (%) | 1 (16.7) | 3 (33.3) | 1 (14.3) | 0 (0) | 0.524 |  |
| Pulmonary vein ablation (%) | 1 (16.7) | 1 (11.1) | 0 (0) | 1 (25.0) | 0.619 |  |
| ER visits for any cause (%) | 3 (50.0) | 5 (55.6) | 3 (42.9) | 2 (50.0) | 0.968 |  |
| Bold values indicated significance at alpha threshold of 0.05.  ER: emergency room, ICD: implantable cardioverter defibrillator, PM: pacemaker. | | | | | | |
